# Histidine to glutamine substitutants, a regulated process that impacts cell survival and suggests histidine shortage in cancer

**DOI:** 10.1038/s41420-026-03225-5

**Published:** 2026-06-27

**Authors:** Domenica Lovecchio, Pierre-René Körner, Lorenzo Valcanover, Onno B. Bleijerveld, Bart R. J. Blokhuis, Abhijeet Pataskar, Jasmine Montenegro Navarro, Demi Wernaart, Liesbeth Hoekman, Daniil Anastasopoulos, Kim Vrijland, Xiaodong Feng, Julien Champagne, Karin E. de Visser, Olaf van Tellingen, Frank A. Redegeld, Reuven Agami

**Affiliations:** 1 Division of Oncogenomics, Oncode Institute, The Netherlands Cancer Institute, Amsterdam, The Netherlands; 2 NKI Proteomics Facility, The Netherlands Cancer Institute, Amsterdam, The Netherlands; 3 Division of Pharmacology, Utrecht Institute for Pharmaceutical Sciences, Faculty of Science, Utrecht University, Utrecht, The Netherlands; 4 Division of Tumor Biology & Immunology, Oncode Institute, The Netherlands Cancer Institute, Amsterdam, The Netherlands; 5 Division of Pharmacology, The Netherlands Cancer Institute, Amsterdam, The Netherlands; 6 Department of Genetics, Erasmus MC, Rotterdam University, Rotterdam, The Netherlands

**Keywords:** Cancer, Cell growth

## Abstract

Cancer-associated genetic, epigenetic, and microenvironmental factors impose shortages of tryptophan and arginine, which induce specific codon reassignments (substitutants) in their proteomes. Whether cancers are deprived of other amino acids is unknown. Histidine is an essential amino acid reported to be low in certain tumor types. Therefore, we investigated the potential for a histidine shortage in cancer. Using cultured cancer cells, we pinpointed histidine to glutamine (H>Q) substitutants as the most pronounced proteomic event following histidine deprivation. We then scanned cancer proteomes for H>Q proteins and observed a marked enrichment in pancreatic, uterine, and kidney cancers. Mechanistically, we propose that H>Q is a result of tRNA misalignment, and used tRNA glutamine (tRNA(Gln)) modifications at the wobble position to demonstrate it. We further show that URM1-mediated U34 thiolation boosts H>Q production and influences the survival of cancer cells following histidine deprivation, suggesting a potential role for H>Q. Additional characterization of H>Q substitutants revealed preferred sequences and the maintenance of H>Q host protein expression despite the absence of histidine, indicating cellular regulation and functional consequences. Thus, histidine shortage induces H>Q substitutants, a regulated mistranslation event that pinpoints histidine limitation in cancer and impacts cell survival.

## Introduction

The cancer proteome is far more intricate than previously understood, encompassing a “hidden” proteome shaped by metabolic reprogramming [1–4]. Altered metabolism is a fundamental hallmark of cancer that evolves during tumor progression, with tumors shifting from oxidative phosphorylation to glycolysis. These metabolic adaptations, driven by oncogenic signaling and the tumor microenvironment, enable cancer cells to sustain rapid proliferation, maintain cellular homeostasis, and evade anti-tumor immune activity [5–7]. However, such metabolic adaptations impact the quality of the cancer proteome.

For example, accelerated tryptophan consumption along the kynurenine pathway suppresses anti-tumor activity and reduces its availability for protein synthesis. Consequently, codon reassignment occurs, generating substitutants -distinct from genetically encoded mutants- in which tryptophan codons are replaced by phenylalanine codons (W>F substitutants) in the cancer proteome [2]. Another example of substitutants occurs under conditions of arginine shortage, in which arginine production is suppressed in many cancer cells, increasing the availability of its precursor, aspartate, for nucleotide production and stimulating cellular proliferation [8]. In lung cancer, limited arginine availability results in high arginine to cysteine substitutants levels, potentially allowing better adaptation to oxidative stress [3].

Arginine shortage further induces a broader spectrum of substitutants (including R>K, R>S, R>H, and R>Q), some of which can be presented as neoepitopes recognized by T-cells, highlighting the immunological relevance of amino acid-driven translational rewiring [9]. Extending this concept, recent observations indicated low histidine levels in certain types of cancers, suggesting that amino acid limitation may occur across distinct metabolic contexts in tumors [10–13]. In addition, histidine restriction has been reported to exert strong antileukemic effects in vivo [14]. Importantly, histidine is the sole precursor to histamine, whose production by mast cells in the tumor microenvironment inhibits the anti-tumor activity of macrophages via histamine receptor H1 (HRH1), thereby playing an important role in cancer immunometabolism [15, 16].

Altogether, these findings connect histidine availability to translational control and metabolic fitness in cancer cells, highlighting how amino acid limitation can directly influence tumor growth.

Here, we show that histidine to glutamine (H>Q) substitutants are the major aberrant protein event induced by histidine shortage. We used this knowledge to scan human cancer proteomes and detected enriched H>Q events in pancreatic adenocarcinoma (PDAC) and other cancer types. These H>Q events are partially regulated by URM1-mediated tRNA glutamine (tRNA (Gln)) modifications at the wobble position [17]. Additionally, we show that H>Q production is mRNA context-dependent, sustains protein production, and affects cell survival under histidine deprivation.

## Results

### Histidine to glutamine (H>Q) substitutants as a marker of histidine shortage in cancer

**Fig. 1:**
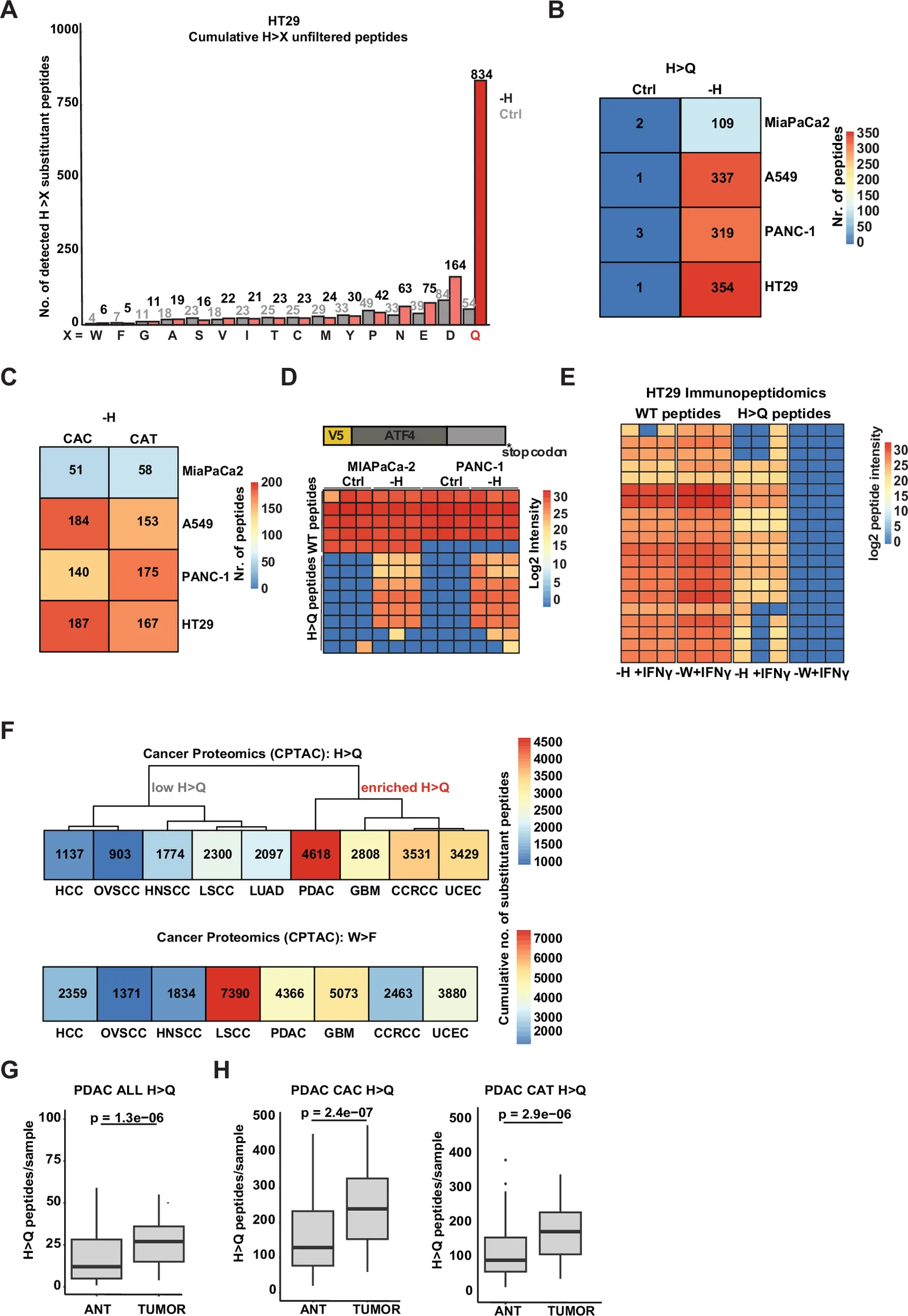
Detection of histidine to glutamine (H>Q) substitutants in cancer proteomics. **A** Barplot depicting the number of histidine substitutants peptides found in HT29 cell line DDA mass spectrometry. Displayed are number of H>X substitution peptides (834 H>Q peptides out of 117,364 total WT peptides) in histidine depleted condition (−H 48 h and 4 h of the proteasome inhibitor MG-132) (in red) and number of background H>X peptides in non-treated condition (Ctrl) (in gray). **B** Heatmap depicting the number of enriched H>Q substitutants peptides expressed in all replicates in histidine depleted condition and the number of background H>Q substitutant peptides expressed in all non-treated (Ctrl) conditions, in four cell lines (MiaPaca2 (DIA), A549 (DIA), PANC-1 (DIA), HT29 (DDA), −H for 48 h except PANC-1 (−H 72 h)). **C** Heatmap depicting the number of enriched H>Q substitutants peptides found in mass-spectrometry expressed in all replicates in histidine-depleted condition (−H), as in 1B separated by histidine codon (CAC, CAT). **D** MiaPaca2 and PANC-1 cells expressing the V5–ATF4 reporter construct (schematically presented) were treated with histidine-depleted condition (−H) for 48 h, MG-132 for 4 h, and then subjected to V5-IP/MS analysis. Heatmap depicts the log2 intensities of H>Q peptides and their corresponding matched WT peptides. Each column represents an independent biological replicate, and each row a separate peptide. **E** Heatmap of immunopeptidomics data in HT29 cells. Displayed are found WT and H>Q substitutant peptides in histidine-depleted (−H) and tryptophan-depleted (-W) conditions with IFN gamma (IFNγ). **F** Heatmap showing the total number of H>Q substitutants peptides in the cancer proteome database (Clinical Proteomic Tumor Analysis Consortium-CPTAC [18]) (upper panel) and of W>F substitutants (lower panel). **G**, **H** Boxplot depicting the cumulative number of H>Q peptides in Tumour and Adjacent Normal Tissues (ANT) of PDAC-CPTAC dataset. In (**G**), all histidine codons were substituted to glutamine. In (**H**), either CAC or CAT codons were substituted to glutamine. P values are calculated by Wilcoxon t-test.

To identify the most prominent mistranslation event that is induced by a shortage of histidine in cancer, we performed mass spectrometry analyses (MS) of HT29 colorectal cancer cells depleted for histidine and analyzed the normal proteome as well as proteomes where every histidine codon (either CAC or CAT) was substituted by any other amino acid (H>X). We did not include any filtering step to unbiasedly detect histidine substitutants (S1A). Figure 1A shows that in the histidine deprivation condition (−H), histidine to glutamine (H>Q) was the most prevalent event, with 834 H>Q peptides detected (compared to 117,364 WT peptides). In control conditions (Ctrl), only 54 H>Q peptides at the background level have been found (Fig. 1A). We validated the induction of high levels of H>Q substitutants following histidine deprivation using a proteomic library where every histidine was substituted with a glutamine (H>Q) across several additional cancer cell lines, including the pancreatic cancer cell lines MiaPaCa2 and PANC-1 (Figs. 1B and S1B). In this analysis, H>Q peptides were filtered to retain only the enriched H>Q peptides after histidine deprivation, and all subsequent analyses used the same filtering steps (see S1A). As histidine is decoded by two codons (CAC and CAT), we also used a proteomics library in which H>Q was generated in a codon-specific manner and found both codons to similarly contribute to H>Q-peptide-induction (Figs. 1C and S1C). To further substantiate this finding, we expressed a V5-tagged reporter and examined MiaPaCa2 and PANC-1 cells undergoing histidine deprivation using V5-IP/MS analysis (Fig. 1D). These experiments further confirmed the induction of H>Q substitutants by histidine depletion in MiaPaCa2 and PANC-1 cell lines (Fig. 1D).

Previously described substitutants, such as tryptophan to phenylalanine (W>F), have been shown to contribute to the surfacesome landscape of cancer cells and be presented to T cells as neoepitopes [2, 3]. To determine whether H>Q substitutants share this property, we performed immunopeptidomics analysis of HT29 cells treated with interferon-gamma (IFN-γ) under either histidine depletion or, as a control, tryptophan depletion to mimic amino acid starvation stress (Fig. 1E). Of note, IFN-γ treatment of HT29 is not sufficient to induce W>F substitutants and requires the addition of depletion of tryptophan, as IFN-γ-mediated IDO1 activation is suboptimal in this cell line. While wild-type (WT) peptides were similarly presented under all tested conditions, H>Q peptides were specifically detected in the condition combining IFN-γ treatment with histidine depletion (Fig. 1E). Thus, histidine shortage enreaches the surfacesome of cancer cells with epitopes containing H>Q substitutants.

We further investigated the dynamics of H>Q accumulation following histidine shortage. We depleted histidine to 50% (48 μM), 70% (29 μM), and 90% (13 μM), and observed a clear and significant increase in H>Q substitutants as histidine shortage increased (S1D, E). Monitoring cell proliferation under varying levels of histidine shortage revealed general growth suppression across all histidine-shortage conditions (S1F). Cells cultured under 50% histidine deprivation continued to proliferate at a reduced rate relative to controls. Additionally, we assayed H>Q substitutants in histidine depletion (48 h) followed by supplementation (+96 μM) for an additional 24 or 48 h (S1D, E). This indicated a significant reduction in H>Q substitutants at 24 and 48 h, with proliferation returning to levels comparable to those in complete medium (Ctrl, S1D, E, G). However, many H>Q substitutants remained, suggesting long-term effects and stability for some of the mutated proteins (S1D). These data suggest a link between histidine shortage, suppression of cell proliferation, and H>Q induction.

### Histidine shortage in cancer proteomes

Next, we explored the potential histidine shortage in physiological conditions in cancer by assessing H>Q substitutants across tumor proteomes from the Clinical Proteomic Tumor Analysis Consortium (CPTAC) dataset [18]. Initially, we utilized a proteomic library in which all histidine codons were substituted with glutamine (Fig. 1F). This analysis identified enrichment of H>Q peptides in pancreatic ductal adenocarcinoma (PDAC), uterine cancer (UCEC), kidney cancer (CCRCC), and glioblastoma (GBM). This group of cancer types is hereafter referred to as “enriched H>Q.” Relatively low H>Q peptides were identified in ovarian (OVSCC), head and neck (HNSCC), liver (HCC), and lung (LSCC and LUAD) cancers (Fig. 1F), hereafter referred to as “low H>Q.” In contrast, W>F substitutants did not display a similar enrichment pattern across the same tumor types. We further compared H>Q peptides in tumors with adjacent normal tissues (ANT) and observed a significant enrichment in PDAC and UCEC (Figs. 1G and S1H left panel). In CCRCC and GBM, we observed a higher or comparable number of H>Q peptides in the ANT compared with the cancer tissue, possibly due to the influence of the adjacent tumor (S1H, right panels). For PDAC, we repeated this analysis using codon-specific H>Q proteomics libraries and observed a similar tumor enrichment phenotype with both CAC and CAT histidine codons (Fig. 1H). This analysis suggests a histidine shortage in several types of cancers.

To investigate whether tumor microenvironment components contribute to H>Q variation, we examined the involvement of mast cells, given their reported role in histidine–histamine metabolism within the tumor microenvironment [15, 19]. We hypothesized that mast cells might contribute to the generation of H>Q substitutants (S2A). Using an in vivo tumor model with altered mast cell composition, baseline H>Q events were detectable in mast cell-deficient tumors (HET), consistent with the physiological occurrence of H>Q substitutants observed in CPTAC datasets (S2B–E). However, H>Q-containing peptides were significantly enriched in tumors from mast cell-proficient hosts (WT), while control peptides (e.g., GAPDH, histones) remained unchanged (S2D–F). These results suggest that mast cell activity can modulate the extent of H>Q substitutants in vivo, although additional factors are likely involved.

To demonstrate a more direct link between mast cell activity and histidine shortage- derived H>Q peptides, we established tissue culture conditions in which human mast cells (HMC1.1) were co-cultured with HT29 cancer cells for 4 or 7 days (S2G). Metabolomic analysis showed a histidine reduction of up to 50% compared to the original media concentration when mast cells (HMC1.1) were co-cultured with HT29 cells (S2H). Encouraged by the 50% reduction in histidine levels, we performed MS of HT29 cells alone and HT29 cells that were co-cultured with HMC1.1 cells and then separated prior to the MS analysis. As shown in S2I, we detected more H>Q peptides in the HT29 co-culture group than in the HT29-only group (S2I). Matched wild-type peptides and control peptides (e.g., GAPDH, histones) showed similar levels across all samples (see S2I, J).

Together, these observations support a model in which microenvironmental factors contribute to histidine limitation and H>Q incorporation in cancer. CPTAC patient tumor samples support the physiological relevance of H>Q substitutants in human tumors, while mast cells may represent one contributing component among additional tumor milieu–dependent factors influencing translational plasticity.

### URM1-mediated tRNA (Gln) modification is required for efficient production of a subset of H>Q substitutants

**Fig. 2:**
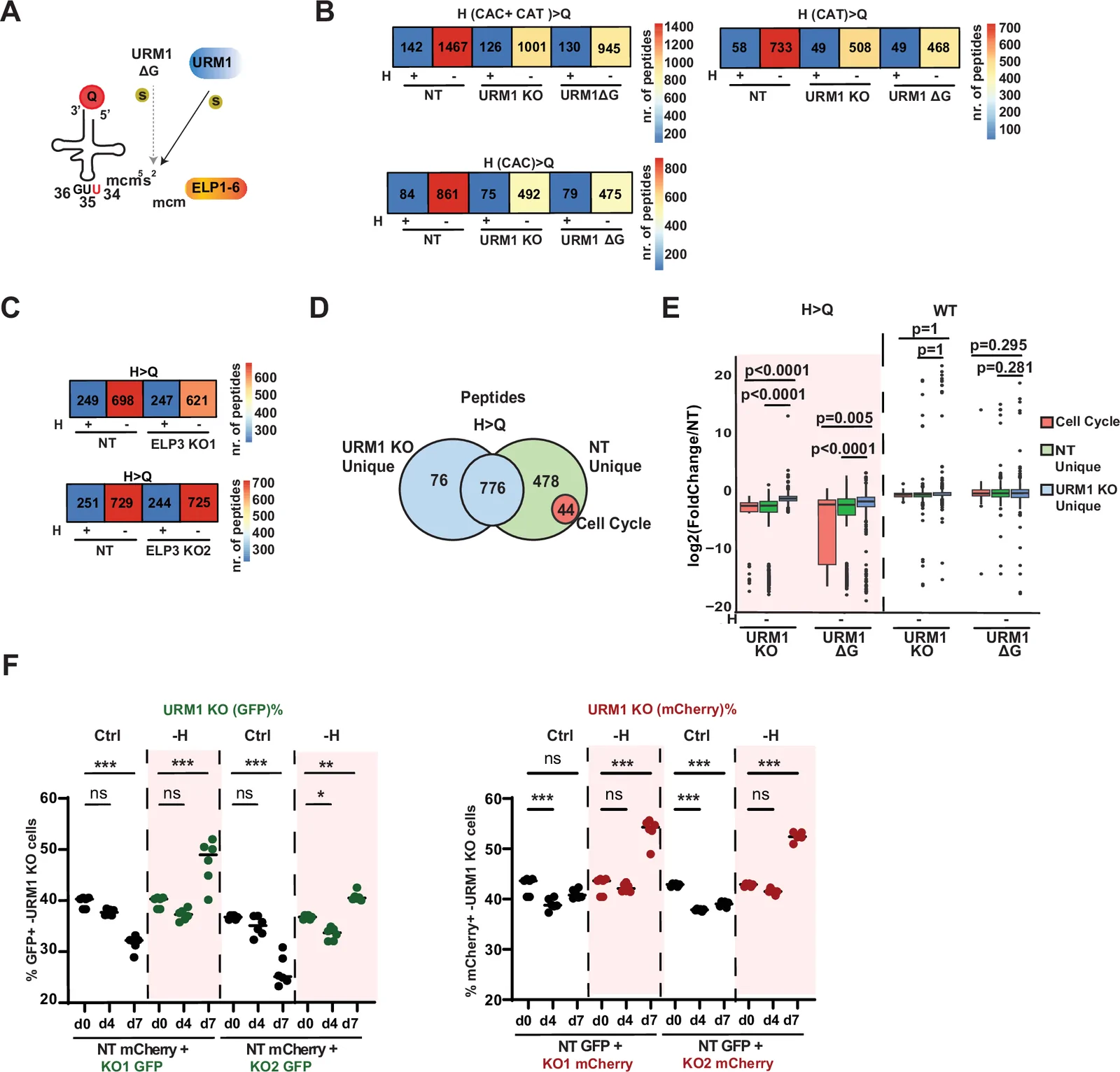
tRNA(Gln) modification by URM1 induces H>Q substitutants and suppresses cell survival to histidine shortage. **A** Schematic representation of tRNA(Gln) mcm^5^s^2^ modification at U34 introduced by URM1and ELPs, enzymes. **B** Heatmaps of DIA proteomics depicting the number of H>Q peptides expressed in all replicates of a condition in PANC-1 cells (in control “+H” or “−H” 72 h, MG-132 for 4 h). Conditions are NT (non-targeting sgRNA), URM1- KO1, and URM1ΔG vector. The panels examine H>Q regardless of the histidine codons, and H(CAC)>Q and H(CAT)>Q histidine codons shown separately. **C** Heatmaps of DIA proteomics depicting the number of H>Q peptides found expressed in all replicates in cells containing ELP3-KOs vectors and subjected to histidine depletion condition (−H 72 h, MG-132 for 4 h). **D** Venn diagram showing the overlap in histidine-shortage-induced H>Q peptides found in PANC-1 cells transduced with either NT (non-targeting sgRNA) or URM1-KO. Green-marked peptides are lost upon URM1-KO. Blue-marked peptides are gained or shared. In red indicated are peptides associated with the GO term cell cycle. **E** In PANC-1 cells. Boxplot showing the log2 fold change in H>Q, and matched WT over the NT protein intensities of URM1-KO and URM1ΔG cells. Colors indicate the groups of overlap; red = “cell cycle”; green = NT unique; blue = URM1-KO unique. The horizontal lines above the boxes indicate the *p* values (Wilcoxon t-test) for the pairwise comparisons. **F** Growth competition assays performed using PANC-1-GFP or PANC-1-mCherry cells with two different URM1-knockouts, compared to a non-targeting (NT) sgRNA control. The assay was conducted under medium conditions involving histidine-depleted (−H) or control (Ctrl). Flow cytometry was used to analyze the cells at 0, 4, and 7 days following treatment. The y-axis represents the percentage of GFP-positive or mCherry-positive cells. Values represent the average of the six independent replicates ±SD. non-significant is marked ns, *p < 0.05, **p < 0.002, ***p < 0.001 by 1-way ANOVA.

Codon usage inspection suggests misalignment of histidine codons at the wobble position (+3) with uridine 34 (U34) of tRNA (Gln) as the mechanism for H>Q substitutions during histidine deprivation (S3A) [20]. To test this notion, we investigated the impact of tRNA(Gln) modifications on H>Q events. We selected modifications that alter only the tRNA wobble position, expecting an effect only on potential tRNA misalignments. The U34 position of tRNA(Gln), located at the anti-codon loop, is modified by both URM1, which adds a sulfur atom, resulting in the formation of 2-thiolated U34, and by the acetyltransferase elongator complex 1-6 (ELP1-6), which adds a 5-carboxymethyl group to form mcm^5^ U34. These modifications are often found together as (mcm^5^s^2^ U34) (Fig. 2A) [21]. ELP1-6 (with ELP3 being the catalytic subunit), and URM1 are crucial for accurate decoding of the genetic code, efficient protein synthesis, and adaptation to different physiological conditions and metabolic states [21, 22]. However, their role in the cancer-driven production of aberrant protein, in general, and H>Q in particular, is unknown.

To investigate the function of URM1 and ELP3, we generated knockout lines in PANC-1 cells. For URM1, we also employed a functional knockout by overexpressing a dominant-negative variant lacking the sulfur donor domain (URM1ΔG), which is incapable of mediating the s^2^U modification (Figs. 2A and S3B, C, and [22, 23]). Both URM1-KO and URM1ΔG cells showed significantly reduced H>Q events, with similar patterns observed for both CAC and CAT histidine codons (Figs. 2B and S3D). Importantly, the number of corresponding WT peptides (S3E) and the intensities of control histone proteins (S3F) remained unchanged. Moreover, WT peptide intensities did not differ across groups, whereas H>Q peptide intensities did (S3G). Consistently, global protein translation rates (S3H) also remained unaltered. A similar impact on H>Q production was obtained in MiaPaCa-2 cells (S4A–C). Loss of ELP3 in PANC-1 cells resulted in a significant, yet only minimal reduction of peptide number (Figs. 2C and S4D, E), with control protein levels (histones) remaining stable across all groups (S4F, G). Altogether, these results suggest that misalignment is the main mechanism of H>Q production and pinpoint the role of U34 tRNA(Gln) mcm^5^ modification in accelerating this process.

### URM1-mediated H>Q substitutants impact the survival of cells during histidine shortage

The impact of URM1-loss on H>Q production is interesting, as it may indicate a potential cellular role. Supporting this notion, when we subjected the group of H>Q proteins that were lost in the URM1-KO condition to GO term analysis, we identified enrichment for cell cycle-related proteins (Figs. 2D and S5A–D). Similar results were observed in proteomics data following ectopic expression of URM1(ΔG) (S5E–H). This suggests a role for URM1 in promoting the translation of H>Q-substituted cell-cycle proteins despite a histidine shortage. To further test this, we examined the fold change in H>Q peptides in URM1-KO and URM1 (ΔG) relative to the NT control. Whereas the expression of the URM1-independent H>Q proteins showed no profound change (Fig. 2E blue group), the URM1-dependent H>Q proteins, including the cell cycle group (red group), were markedly repressed (Fig. 2E green group). No significant difference was observed in the 5′ UTR, CDS, 3′UTR length, or GC content across URM1-KO unique, NT unique, and H>Q common groups (S6A), nor across URM1 (ΔG) unique, NT unique, and H>Q common groups (S6B). Thus, these results suggest that in a histidine-restricted environment, URM1 preferentially promotes translation of H>Q-substituted cell cycle genes, independent of transcript length or GC content.

We further assessed the impact of URM1-loss on cell survival following histidine deprivation using growth competition assays (Fig. 2F). In these experiments, URM1-KO and non-targeting (NT) control cells were co-cultured at nearly equal fractions, and the proportion of URM1-KO cells was monitored over time using flow cytometry (Fig. 2F). We performed this experiment in two ways, marking the URM1-KO cells either with green or red fluorescent proteins to avoid bias. Cells were first treated with either control or histidine-depleted medium for 5 days, then with normal medium for the rest of the experiment. Regardless of the fluorescent marker, on day 7, the URM1-KO cells showed a significant and profound growth advantage compared to the NT control in histidine depletion, whereas their numbers decreased in cells subjected to the control medium during the entire cell growth, as already reported (Fig. 2F, [24]). Importantly, URM1-KO did not affect cells when they were deprived of the essential amino acid tryptophan (S6C). While not excluding indirect effects, the specific survival benefit of URM1-KO cells in a histidine-deprived environment suggests that H>Q substitutions repress the function of cell-cycle proteins, thereby reducing cell survival following histidine deprivation.

Together, our findings support a model in which URM1-dependent U34 modification enables efficient H>Q substitutions under histidine-depleted conditions, particularly affecting proliferation.

### H>Q substitutants maintain host gene expression during histidine shortage

**Fig. 3:**
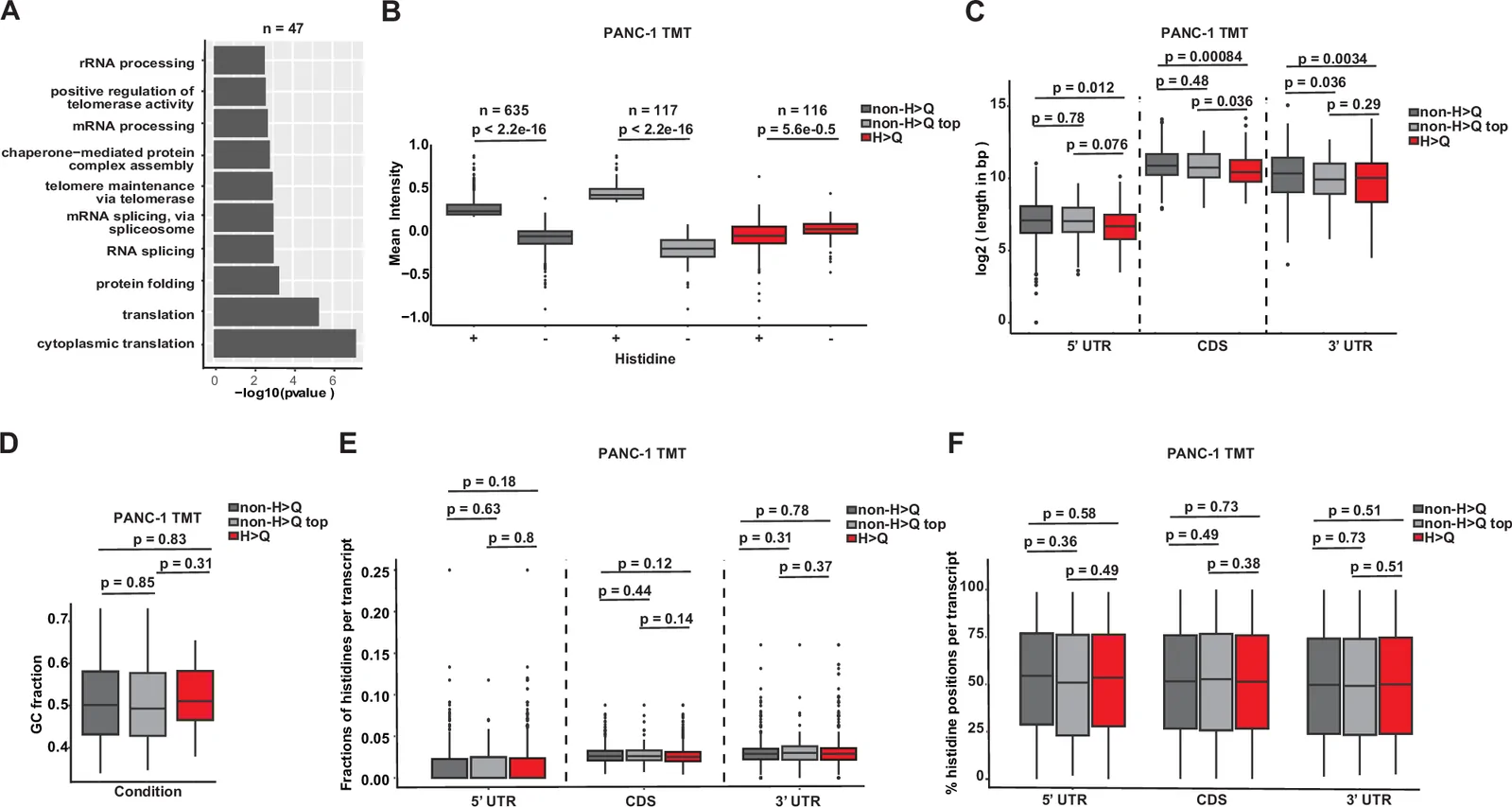
Effect of H>Q substitutions on protein levels. **A** Barplot of Gene Ontology (GO) terms and their *p* values, represented as −log10, sorted with the most significant terms at the bottom. Obtained from the 47 H>Q proteins shared in MiaPaca2 (DIA), A549 (DIA), PANC-1 (DIA), and HT29 (DDA) cell lines (datasets as in Fig. 1B). **B** Boxplots of log2-transformed mean intensity for the three protein groups (indicated by color) in PANC-1 cells, comparing their pairwise conditions of control and histidine depletion (−H 72 h, MG-132 for 4 h) followed by TMT mass-spectrometry. The number of proteins the analysis is based on is indicated above each boxplot group (*n*=nr proteins). The horizontal lines within the boxes represent the mean of each group. **C** Boxplots presenting the log2 length of the 5′ UTR, CDS, and 3’ UTR for each group in PANC-1, separated by line color. **D** Boxplot of GC fraction in the CDS for each group in PANC-1 cells. **E** Boxplot of occurrence of histidine codons displayed as a fraction of all codons per transcript for 5′ UTR, CDS, and 3’ UTR for each group in PANC-1 cells. **F** Boxplot of histidine positions as a percentile of total length in transcripts for 5′ UTR, CDS, and 3’ UTR for each group in PANC-1 cells. The horizontal lines above the boxes in panels B-F indicate the *p* values (Wilcoxon t-test) for the pairwise comparisons.

To further elucidate the effect of H>Q substitutants on the proteins in which they occur, we characterized H>Q events by gene ontology (GO). To do this, we assigned the H>Q peptides to their host proteins and performed an overlap analysis of proteomics datasets from four cell lines (HT-29, MiaPaca2, PANC-1, and A549) derived from various origins (colorectal, pancreatic, and lung cancers). This analysis identified mRNA translation as the main process among the 47 shared proteins of H>Q peptides across the datasets (Fig. 3A; datasets in Fig. 1B). We then divided the expressed proteome into three groups: those that show evidence of H>Q expression (the “H>Q” group) and those that did not show any evidence of H>Q substitutants (the “non-H>Q” group). Since H>Q proteins in the non-TMT datasets (DIA and DDA), displayed high protein intensity (likely due to MS limitations), we also generated a control “non-H>Q” group containing proteins with the highest protein intensity (“non-H>Q top”) to circumvent describing effects that are due to high expression level of the host genes for all the analyzed datasets–TMT included (S1A). Following histidine deprivation, while the protein intensities of the “non-H>Q” and “non-H>Q top” groups were significantly reduced, the abundance of the H>Q proteins was strikingly maintained (Figs. 3B and S7A–E). We further examined protein abundance variation using different proteomic approaches (Tandem Mass Tag-TMT, Data-dependent Acquisition-DDA, or Data-independent Acquisition-DIA) and observed similar results, although Tag-TMT demonstrated greater sensitivity to changes in translation between control and histidine-deprived conditions (Figs. 3B and S7D, E).

To further assess whether H>Q substitutants are associated with specific functional programs, we performed complementary gene ontology (GO) analyses at both the transcriptomic and proteomic levels under histidine deprivation, using tryptophan deprivation as a control for general amino acid stress responses at the transcriptional level. RNA-sequencing analysis (RNA-seq) in HT29 cells revealed that histidine depletion was associated with enrichment of pathways linked to cell migration, cytokine production, and immune regulation, indicative of a more invasive and stress-adapted phenotype, while no enrichment of translation-related processes was observed (S8A–D). Consistently, proteomic GO analysis comparing histidine-deprived and control conditions in the PANC-1 and HT29 TMT datasets did not identify translation-related pathways as globally upregulated. Instead, stress response pathways were prominently enriched under histidine deprivation, whereas downregulated proteins were associated with cell division and proliferation, in line with the reduced growth observed under these conditions (S1F, G and S9A–D). In contrast, translation-related pathways were specifically enriched when restricting the analysis to proteins containing H>Q substitutants in both cell lines, and were not detected in the corresponding non-H>Q protein groups (S10A–D). These findings suggest that the maintenance of translation-associated functions is a distinctive feature of the H>Q-containing proteome under histidine limitation.

Additional analysis of mRNA features indicated that maintaining H>Q protein expression during histidine depletion was associated with a slightly reduced coding sequence (CDS) length in the PANC-1 TMT dataset (Fig. 3C). This difference was not consistently observed across all datasets and, therefore, was not considered relevant to H>Q protein expression (S11). Furthermore, no difference was observed in 5′UTR or 3′UTR length or in the GC content of the CDS (Figs. 3C, D and S11). The fractions of histidine residues per protein, or their percentage, were also stable (Fig. 3E, F). Importantly, histone protein expression remained constant across the various conditions (S12 and S13). Altogether, these findings point to H>Q substitutions as a potential cancer-specific adaptive mechanism that preserves translation-associated functions during histidine limitation.

### Context-dependent production of H>Q substitutants

**Fig. 4:**
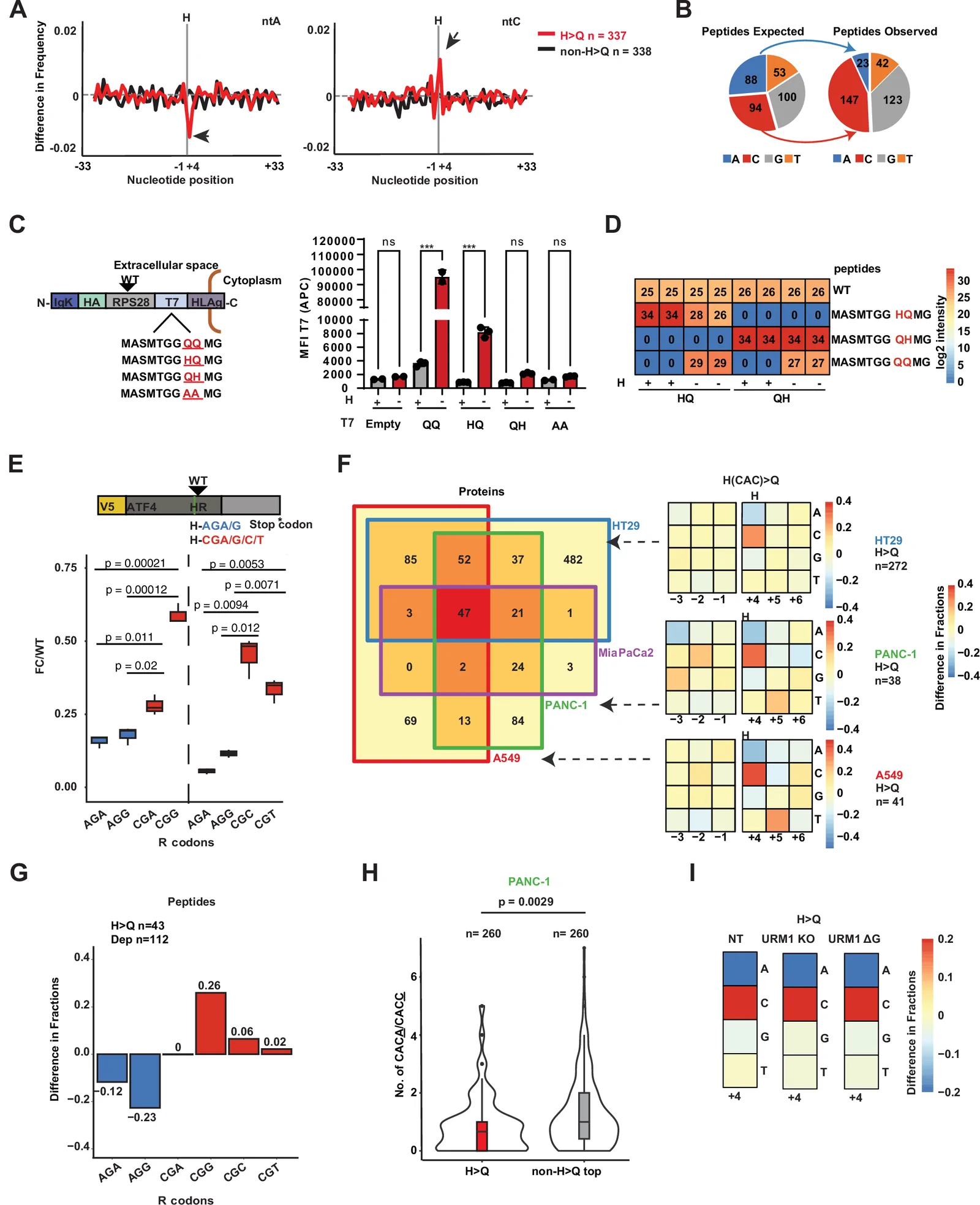
Nucleotide signature of H>Q events. **A** Line Plot of the difference in nucleotide frequency in the A549 (DIA) dataset (−H 48 h, MG-132 for 4 h) along a window of 33 nucleotides before and after the histidine codon (marked by a dashed line). H>Q peptides are displayed in red, and depleted control peptides (non-H>Q containing peptides) in black. **B** Circle diagram of expected and observed nucleotide frequencies around histidine in the A549 (DIA) dataset (−H 48 h, MG-132 for 4 h). **C** The left panel shows a schematic of the T7 tag reporter with its mutant variants, highlighting parts of the QQ (CAG CAA), HQ (CAT CAG), QH (CAG CAT), and AA (GCT GCT) amino acid sequences used in this study. H = histidine, Q = glutamine, A = alanine. The right panel shows flow cytometry analyses in MD55 cells under the indicated conditions (“Empty,” “QQ,” “HQ,” “QH,” “AA”) in the presence (+H) or absence (−H) of histidine. Values represent the mean of three independent replicates ± SD. Values represent the average of the three independent replicates ±SD. MFI = mean fluorescence intensity, APC = fluorescence label for T7 detection. ns non-significant *p < 0.05, **p < 0.002, ***p < 0.001 by 1-way ANOVA. **D** Heatmap of the log2 Intensity of T7 reporter expression (as in **C**, T7 QQ, T7 HQ, and T7QH) measured by HA-IP/MS of T7 HQ and T7 QH in MD55 transduced cells, in control (+H) or histidine shortage conditions (−H 48 h, MG-132 4 h). WT peptide is a control peptide, part of the histidine-free protein RPS28 (scheme **C**). **E** In the upper panel, schematic of the reporter constructs (V5-ATF4-HR) used to detect H>Q events, featuring six different versions of the codon R: AGA, AGG, CGA, CGG, CGC, and CGT. H = histidine, R = arginine. In the lower panel boxplots showing the fold change of H>Q peptide intensity over the endogenous peptides of V5-IPed reporters (WT) in HT29 cells that were also subjected to histidine shortage for 48 h and 4 h of MG-132 treatment. The dashed line separates two individual experiments. Each reporter contains one histidine codon followed by a different arginine codon, as indicated in the x-axis. The horizontal lines above the boxes indicate the *p* values (Wilcoxon t-test) for the pairwise comparisons. **F** Venn diagram showing the overlap in H>Q peptides detected in different datasets, as in Fig. 1B. The three groups of unique peptides in HT29 (DDA), PANC-1 (DIA), and A549 (DIA) were investigated in more detail regarding their heatmap signatures following the CAC codon. The number of CAC histidine codons and the cell line the analysis is based on is indicated next to each heatmap (*n*=nr codons). **G** Barplot of difference in arginine codon frequency after histidine CAC codon in the proteomic datasets described in HT29 (DDA), PANC-1(DIA), and A549 (DIA) datasets (same as in Fig. 4F). **H** Violin plot of the CACA/CACC ratio for the two protein groups (color-coded) in the PANC-1 DIA dataset (same as in Fig. 4F). Horizontal lines show group means. *P* values by Wilcoxon t-test for the pairwise comparisons. **I** Heatmaps of differences in nucleotide frequency on position +4 after Histidine (CAC) (H = 0, +3) showing the groups NT, URM1 KO, and URM1 ΔG, compared against expressed wild type, dataset as in Fig. 2B.

Our results above suggest that H>Q substitutants are produced by specific tRNA misalignment events at the wobble position of histidine codons (Figs. 2 and S3A), and that proteins with H>Q incorporations remain relatively abundant under histidine-deprived conditions. We therefore speculated that surrounding mRNA sequences near H>Q positions may influence the efficiency of their production during histidine shortage. To examine this issue, we compared the frequency of each nucleotide in the vicinity of histidine between regions with evidence for H>Q events and those without. We first used the enriched H>Q peptide list (S1A), extracted the frequency of each nucleotide surrounding (∓33 nt) each histidine codon, and compared it to the normal frequency of the nucleotides in the expressed proteome of the cell line. This analysis indicated a profound bias toward a single position (+4, the first nucleotide following the wobble position of the histidine codon, Fig. 4A). In this position, we observed a reduced presence of A and an increased presence of C (“H>Q” group). In contrast, a similar search using a random set of histidine codons that did not show H>Q production did not yield any bias, particularly at position +4 (“non-H>Q” group, Fig. 4A). The changes in nucleotide A and C frequencies were profound, as they accounted for 60–75% of the expected events (Fig. 4B); A decreased from 88 to 23 (74% reduction), and C increased from 94 to 147 (60% increase). In contrast, the nucleotides G and T showed a limited difference of ~20% at this position (Fig. 4B). Moreover, a similar analysis using W>F peptides arising from mis-aminoacylation under tryptophan depletion, rather than from codon misreading, did not produce such a signature (S14A, [2]). To determine whether the observed pattern reflected a nucleotide-level bias rather than an amino acid–level bias, proteomics data were analyzed, revealing no significant differences in amino acid composition (S14B).

To validate the bias in H>Q production following histidine deprivation, we designed a specific H>Q reporter vector for flow cytometric analysis that allows changes in the residues surrounding histidine. This reporter contained a histidine-free sequence, consisting of an IgK leader, an HA-tag for HA-IP/MS, the RPS28 protein, and a T7-tag epitope with two glutamine residues (MASMTGGQQMG), which was expressed at the surface of transduced cells via a transmembrane domain from the HLA-G gene to ensure membrane localization (Fig. 4C, left panel). Upon transduction, the T7 tag is exposed on the surface of the transduced cells, allowing quantification by flow cytometry using an anti-T7 antibody. To detect H>Q events, we mutated glutamine residues (QQ) to histidine in two T7 reporter constructs: T7HQ and T7QH (Fig. 4C). As a control, both glutamines were mutated to alanine in a T7AA construct (Fig. 4C). Importantly, while the histidine residue in T7HQ was followed by a C nucleotide in the context of T7QH, it was followed by an A. We therefore hypothesized that if H>Q bias is real, we should detect more H>Q in the context of T7HQ than T7QH.

Flow cytometric analysis showed a high level of T7 signal for the T7QQ reporter, which increased further under histidine shortage (Fig. 4C, right panel), likely due to its accumulation at the cell membrane resulting from the proliferation arrest (S1F, G). T7HQ, T7QH, and T7AA, on the other hand, did not show any signal above background (Fig. 4C, right panel). However, under histidine-deprivation conditions, we observed a significant level of T7 expression in the T7HQ vector, whereas the T7QH construct showed no significant signal above background (Fig. 4C, right panel). This difference was verified by HA-IP/MS (Figs. 4D and S14C). While we observed similar expression of both reporters under control and histidine-depletion conditions (as determined by quantification of the log2 intensity of a T7-unrelated vector peptide in the RPS28 protein, named WT, Fig. 4C, D), and similar expression of the T7HQ and T7QH peptides under control conditions (+H, Fig. 4D), following histidine deprivation, the conversion of HQ to QQ was significantly more pronounced than that of QH to QQ (−H, Fig. 4D). Using the raw values, we calculated fold changes for HQ and QQ relative to their WT counterparts to quantify the expression differences. This resulted in an expression difference of ~2-fold (log2((4.09–2.98)) without normalization, or a relative 166-fold difference when normalized to the levels of the parental HQ and QH peptides (log2(4.09–2.98) − (1.82–8.09)) = 7.38, Fig. 4D). Clearly, while the fraction of QH>QQ protein was minor and did not affect the amount of the parental protein in the sample (as shown from the expression of the QH peptide in the HA-IP, log2 34), the production of HQ>QQ protein was so high that it markedly reduced the fraction of the parental HQ protein in the sample (Fig. 4D). Importantly, control experiments assessing mRNA expression levels of the T7HQ and T7QH constructs showed no significant difference, ruling out expression bias as a cause for the differential substitution efficiency (S14D). Thus, this result is consistent with the biased production of H>Q at the +4 nucleotide (accelerated production in C compared with A).

To further pinpoint the requirement of the +4 nucleotide, rather than a particular amino acid, we focused on histidine followed by arginine (HR), as arginine is encoded by six codons - two of which start with an A (AGG, AGA) and four start with a C (CGA, CGG, CGC, CGT). V5-IP/MS analysis of cells transduced with the various V5-ATF4-HR vectors showed enriched HR>QR vs WT expression in all the C arginine codons compared with the A arginine codons following histidine starvation (Figs. 4E and S14E). Together, these findings confirm a nucleotide-level bias rather than an amino acid–level effect (Figs. 4E and S14E). We next controlled the origin of the nucleotide signature and found no global sequence bias that could explain the overrepresentation of A- and C-starting codons at the +4-position following the H(CAC) codon (S15A, B). In contrast, for the H(CAT) codon, a positional bias became apparent, with H(CAT)-G combination being overrepresented relative to H(CAT)-A (S15A, B). Altogether, these results experimentally validate the signature of H(CAC)>Q efficiency.

### H>Q signature contributes to protein expression in histidine-deprived conditions

Sequence biases in codon reassignments were not observed before. Hence, we inquired whether the H>Q signature is driven by a set of abundant sites or is an intrinsic property of these substitutants (S15C, D and S16). For this analysis, we focused on the CAC codon because its signature was most pronounced (S15 and S16), and we examined the overlap of H>Q proteins across the various cell lines (colorectal, pancreatic, and lung cancer, as in Fig. 3A). While this analysis identified 47 H>Q proteins shared across all these proteomics datasets, a large proportion of proteins were uniquely present in a single cell line (Fig. 4F); e.g., HT29 (482 proteins), PANC-1 (84), and A549 (69 proteins). We therefore repeated the frequency analysis using the H>Q peptides from the unique protein groups of HT29 (272 peptides), PANC-1 (38 peptides), and A549 (41 peptides), and obtained a consistent signature at the +4 position in all cases (Fig. 4F). These analyses indicate that the H>Q signature at the +4 position is an intrinsic feature of H>Q substitutant production, not biased by the selected H>Q protein set.

To further validate H(CAC) signature, we analyzed several proteomes (HT29-, MiaPaCa2-, PANC-1-, and A549- DDA) for all the H(CAC)R>QR proteomic events and binned them into the different arginine codons (Fig. 4G). Compared with their normal frequency of appearance in the expressed proteome, the arginine codons AGG and AGA showed reduced frequency, while the CGC, CGG, CGA, and CGT showed increased abundance, consistent with Fig. 4E.

Finally, because H>Q maintains protein expression under histidine deprivation, generating mutated proteins, we asked whether the identified H>Q sequence signature at position +4 contributes to this phenomenon. We, therefore, compared the “H>Q” and “non-H>Q top” protein groups—the same groups as in Fig. 3—by the ratio of CAC codons followed by A versus C. In line with our assumption and across all datasets, the H>Q group consistently showed a significant reduction in the CACA/CACC ratio compared with the non-H>Q group (Figs. 4H and S17A–E). Moreover, GO term analysis of the highest decile of H>Q proteins revealed enrichment for mRNA translation pathways, as in Fig. 3A, supporting the conclusion that CACC sequences enhance translation of H>Q proteins relative to CACA sequences (Fig. S17F). Thus, H>Q sequence preference appears to contribute to protein expression in histidine-deprived conditions (Figs. 3, 4H and S17A–F). Notably, despite the increase in H>Q abundance, URM1 activity did not influence the observed signature (Figs. 4I and S17G).

Overall, our data show that histidine deprivation induces H>Q substitutants by a tRNA misalignment mechanism. Consequently, H>Q production is determined by at least two factors. tRNA modifications that affect global H>Q expression, and mRNA sequences (particularly at the +4 position relative to the histidine codon) that induce the production of H>Q at preferred sites. Generally, H>Q substitutants allow the production of selected sets of proteins, with consequences for their function and cell survival.

## Discussion

In this manuscript, we investigated histidine shortages in cancer by interrogating aberrant mRNA translation. We first defined histidine to glutamine (H>Q) substitutants as the primary mistranslation event following histidine depletion in cultured cancer cell lines. We then used this knowledge to pinpoint histidine shortage across multiple cancer patients’ datasets, with particularly high H>Q levels in pancreatic cancer. Further characterization of the histidine shortage-induced H>Q events revealed an impact on gene expression that affects cell survival in histidine-deprived conditions. Lastly, we demonstrate that the H>Q generation is a regulatory event involving both tRNA modifications and mRNA signatures.

### Substitutants as a method to detect amino acid shortages in cancer

In previous work, we anticipated shortages of tryptophan and arginine in cancer, based on known links to metabolic pathways and amino acid measurements [25–27]. While histidine is an essential amino acid in humans, cancer-related pathways that increase histidine demand have not been described. As amino acid shortages depend on supply and demand, they are notoriously challenging to pinpoint directly. We exploited the cancer-associated mistranslation process that characterizes amino acid-deprived cancer cells to identify amino acid shortages that are not readily apparent in gene expression and metabolomics cancer datasets [2, 3, 9]. Indeed, our investigation proposes H>Q substitutants as a unique biomarker of histidine deficiency and has pinpointed their occurrence in several cancer types. Thus, well-defined aberrant protein expression patterns can be utilized to identify amino acid shortages in cancer.

### tRNA modifications as regulators of mistranslation

Our investigation into the production of H>Q substitutants revealed a surprising role of a tRNA(Gln) modification in stimulating this process. Codon usage inspection suggests that H>Q arises from a mismatch in the alignment of tRNA(Gln) and histidine codons at the wobble position [20]. Traditionally, mcm^5^s^2^ tRNA modification at the anticodon wobble position is associated with enhanced decoding of codons enriched in lysine, glutamine, and glutamate, helping cells adapt to metabolic stress [12, 26–28]. While normally low in healthy tissues, U34-modifying enzymes are elevated in many cancers, supporting cancer stem cell maintenance and metastasis by boosting translation of stress-adaptive proteins and contributing to proteome stability [22]. However, their involvement in amino acid substitutions had not been previously investigated [28–31]. Our results suggest that elevated levels of U34 modifications increase aberrant mRNA translation in cancer cells with phenotypic consequences.

By directly examining aberrant protein translation of histidine codons, we show that URM1-mediated thiolation of U34 tRNA(Gln), but to a lesser extent ELP3-mediated carboxy-methylation of the same tRNA site, stimulates aberrant protein production – enhancing glutamine incorporation. In addition to tRNA(Gln), URM1 thiolates tRNA(Lys) and tRNA(Glu) in all eukaryotes [17]. An outstanding question is whether this unexpected function of URM1 is confined to H>Q substitutants or reflects a more general mechanism driven by URM1-dependent modifications of lysine- and glutamate-tRNAs that promote additional tRNA–codon misalignment events.

These observations raise the possibility that URM1-dependent translational plasticity may represent a therapeutically exploitable vulnerability in cancer.

### Substitutants, a way to control cellular responses to amino acid shortages

The boosting effect of URM1 on the production of H>Q substitutants may imply a function during metabolic stress periods. More broadly, cellular responses to amino acid limitation are typically coordinated by established pathways such as the integrated stress response (ISR), amino acid-sensing mechanisms, and endoplasmic reticulum (ER) stress signaling, which generally act to reduce global translation while promoting adaptive gene expression programs [32, 33]. In this context, we found that H>Q events lead to sustained protein production during histidine deprivation and that URM1 expression boosts H>Q production in a subset of proteins with cell-cycle-regulating functions. Notably, our analyses did not reveal consistent involvement of H>Q-incorporating proteins in the processes or pathways of canonical ISR activation, amino acid sensing, or ER stress signatures across cell lines, supporting the conclusion that the observed effects do not strictly rely on these pathways. At the same time, URM1 thiolation impaired cell survival during histidine shortage. This impairment in cell survival suggests that high levels of H>Q-substituted protein groups, including those translated from cell-cycle genes, impact recovery following stress. In contrast, in the absence of URM1, mainly WT cell cycle proteins are present, which may support a more functional cell cycle and confer a growth advantage independent of histidine shortage.

### mRNA signatures of amino acid substitutants

Our experiments indicated that H>Q substitutants favor a C and disfavor an A at the first nucleotide following the codon (position +4). We report that this signature is an intrinsic property of H>Q events, observed across different cell lines and gene groups, and is not affected by the URM1-mediated H>Q boosting effect. Moreover, no such nucleotide signature was observed in sites that generate W>F substitutants following tryptophan depletion. This is explainable, as misalignment between tRNA and mRNA is suggested to be the cause of H>Q substitutants, while mis-aminoacylation at the tRNA-charging step is the prime cause of W>F [2, 20]. The impact of H>Q sequence preference on protein expression during histidine shortage underscores its functional relevance (Fig. 4H). However, the underlying mechanism for the precise nucleotide preference of H>Q remains elusive. Future experiments that examine in detail additional misalignment events, as well as biochemical and structural data of codon/anti-codon interactions, may shed light on the causes of this nucleotide signature (Fig. 4).

### Potential exploitation of H>Q substitutants for cancer therapy

The expression of H>Q proteins may be exploited as a cancer vulnerability in at least two ways. First, we observed that high H>Q protein production compromises cell viability, potentially through excessive accumulation of substitutants in key cell-cycle proteins. This suggests that further induction of histidine deprivation in cancers with limited histidine availability, such as PDAC, might inhibit tumor progression. Indeed, a histidine-based dietary approach was recently reported to extend survival in leukemic mice in a dose-dependent manner while remaining well tolerated [14]. This highlights the potential of histidine manipulation as a tolerated intervention in cancer models. Moreover, it also provides new insights into studies supporting histidine restriction as a complementary strategy alongside chemotherapy [11, 34, 35].

Our findings further identify URM1-dependent tRNA thiolation as a key regulator of H>Q production, linking translational plasticity to metabolic adaptation. Its dependence on sulfur transfer suggests that cellular sulfur availability may serve as an indirect means of modulating tRNA thiolation and H>Q levels [17, 21], thereby coupling metabolic state to the extent of adaptive mistranslation.

Second, in cancer types with poor prognosis, such as PDAC, histidine shortage can lead to the production of H>Q proteins, which may influence cancer cell proteome (see Fig. 1). This alteration could potentially enhance the susceptibility of these cells to new immunotherapeutic strategies, as recently shown for W>F, R>S and R>K substitutants, positioning them as a promising strategy for targeting tumor cells [36, 9]. Importantly, increased URM1 activity under histidine limitation may further enhance H>Q incorporation, particularly in cell-cycle proteins, thereby coupling reduced proliferative capacity with increased tumor immunogenicity.

Together, these observations suggest that H>Q substitutants represent a potential vulnerability linking metabolic stress, translational control, and immune recognition in cancer. Stress-adapted cancer cell subpopulations, such as drug-tolerant persister or tumor-initiating cells, may represent a relevant context in which H>Q-mediated translational plasticity could operate, although this remains to be determined.

## Materials and methods

### Cell lines and reagents

The MD55A3 cell line was derived from metastatic melanoma tumor resections collected with informed patient consent under a protocol approved by the National Institutes of Health (NIH) IRB Ethics Committee and approved by the MD Anderson IRB (protocol numbers 2012-0846, LAB00-063, and 2004-0069; NCT00338377) [4]. MD55A3 were cultured in Roswell Park Memorial Institute 1640 Medium (RPMI 1640, GIBCO) supplemented with 10% heat-inactivated fetal bovine serum (Sigma), 25 mM HEPES (GIBCO), and 100 U ml^−1^ penicillin/streptomycin (GIBCO). All the other cancer cell lines originated from the American Tissue Culture Collection (ATCC) and were grown in the recommended culture media. In depth, A549, HT29, MiaPaCa 2, SW480, and PANC-1 cells were cultured in Dulbecco’s modified Eagle’s medium (DMEM; GIBCO), supplemented with 10% fetal bovine serum (Sigma) and 100 U/ml penicillin–streptomycin (GIBCO). Aspc-1 were cultured in RPMI 1640 (GIBCO) supplemented with 10% fetal bovine serum (Sigma) and 100 U/ml penicillin– streptomycin (GIBCO). All cell lines were maintained in a humidified atmosphere containing 5% CO2 at 37 °C. All cell lines were tested regularly and were found to be negative for mycoplasma contamination (EZ-PCR mycoplasma kit; Biological Industries).

To induce specific amino acid depletions in cells, special media were prepared, lacking the amino acid of interest. Histidine-free medium was prepared by dissolving 7.4 g of “RPMI 1640 modified medium w/o L-Glutamine, amino acids and glucose” (USBiological) and 2 g of sodium bicarbonate (Sigma) in 800 mL of demineralized water. Next, 10 mL of 200 g/L glucose (GIBCO) and the different amino acids were added, excluding L-Histidine. The pH was then adjusted to a range of 7.2–7.4, and demineralized water was added to reach a total volume of 1 liter. Finally, the medium was filter-sterilized using a “Stericup® Vacuum Filter” (0.22 μm pore size, Millipore).

Tryptophan-depleted medium was prepared by dissolving 12.048 g of “DMEM F-12 w/o Tryptophan (Powder) (USBiological)” and 1.2 g of sodium bicarbonate (Sigma) in 800 mL of demineralized water.

The pH was then adjusted to a value of 7.2, and the medium was filter-sterilized with a “Stericup® Vacuum Filter” (0.22 μm pore size, Millipore). Before use, both types of amino acid-depleted medium were supplemented with penicillin/streptomycin (100 U/mL) and 10% dialyzed fetal bovine serum (GIBCO), which was previously heat-inactivated for 20 min at 56 °C. G-132 (Selleckchem) was dissolved in dimethyl sulfoxide (DMSO) and used at a final concentration of 10 μM for 4 h before sample’s harvesting. IFNg (PeproTech) was used at 250 U/ml for 48 h. Polyethylenimine (PEI, Polysciences) was dissolved in water at concentration of 1 mg/ml.

### Plasmid constructs

The V5-ATF4 construct was generated as previously described in [2]. Gene blocks (Integrated DNA Technologies) were synthesized for the V5-ATF4 HR:AGA/CGC/CGT tGFP constructs containing only one histidine per construct (Supplementary Table S1). These sequences were digested/ligated in the XbaI and NotI sites in the pCDH-Blast vector previously described in [2] using T4 DNA ligase (Thermo Scientific) for 1 h at room temperature. The ligation products were subsequently transformed to DH5α-competent bacteria (Invitrogen). V5-ATF4 HR:AGG/CGA/CGG tGFP were obtained by PCR amplification of the plasmid V5-ATF4 HR AGA with XbaI-V5 fw primer and HR(AGG)/HR(CGA)/HR(CGG) reverse primers (Supplementary Table S1). PCR products were extended with tGFP by a second PCR with V5-ATF4 HR:AGA as a template and Not-tGFP rev primer (Supplementary Table S1). The V5-ATF4 HR AGG/CGA/CGG tGFP genes were then digested and inserted in the pCDH Blast vector by restriction/ligation cloning in the XbaI and NotI sites. The ligation products were subsequently transformed to DH5α-competent bacteria (Invitrogen). For the T7 reporter cloning, the DNA sequence coding for IgK-HA-RPS28 was ordered as a gBlock (Supplementary Table S1). Subsequently, the region coding for T7(QQ)-HLAg was added through PCR, and the whole construct was cloned in a pCDH-Blast backbone by restriction-ligation in the XbaI and NotI sites. Starting from this product, the different versions of the T7 tag (QQ, HQ, QH, AA) were introduced by PCR, using the primers listed in Supplementary Table S1 and subsequent restriction-ligation cloning in the XbaI and XhoI sites.

For the IP-optimized T7 construct, an additional arginine residue was introduced in the T7-HLAg linker region by PCR using the primers listed (Supplementary Table S1), and subsequent restriction ligation was cloned into the pCDH-Blast backbone in the XhoI and NotI sites. The ligation products were subsequently transformed to DH5α-competent bacteria (Invitrogen).

For CRISPR-Cas9 cloning, the pLentiCRISPRv2-puro plasmid was digested using FastDigest Esp3I and dephosphorylated with FastAP enzymes (both from Thermo Scientific). Then, the digested vector product was run on a 1% agarose (Merck) gel and was purified using the “Wizard SV Gel and PCR clean-up system” (Promega). Oligonucleotides targeting URM1 and ELP3 (Supplementary Table S1) were annealed and phosphorylated using T4 PNK. The digested vector and the annealed oligos were ligated using T4 DNA ligase (Thermo Scientific) for 1 h at room temperature. Finally, the reaction product was used to transform DH5α-competent bacteria (Invitrogen). A gBlock was synthesized for URM1 (Supplementary Table S1). URM1 was subsequently amplified with the primers XbaI-URM1 Fw and BamHI-URM1 Rev or BamHI-URM1 (ΔG) Rev (Supplementary Table S1). These sequences were digested/ligated in the XbaI and BamHI sites in the pCDH-Blast vector using T4 DNA ligase (Thermo Scientific) for 1 h at room temperature. The ligation products were subsequently transformed to DH5α-competent bacteria (Invitrogen). All resulting plasmids were sequence verified by Sanger sequencing (Macrogen).

### Lentiviral production and transduction

For lentivirus production, 4 × 10^6^ HEK293T cells were seeded per 10 cm dish, 1 day before transfection. For each transfection, 10 μg of the pCDH reporter, 5 μg of pMDL RRE, 3.5 μg of pVSV-G, and 2.5 μg of pRSV-REV plasmids were mixed in 500 μl serum-free DMEM. Next, 500 μl of serum-free DMEM containing 63 μl of a 1 mg/ml PEI solution was added. The entire mix was vortexed and left for 20 min at room temperature, after which it was added to the HEK293T cells to be transfected. The next day, the medium was refreshed, and the lentivirus-containing supernatants were collected 48 and 72 h post-transfection, and snap-frozen in liquid nitrogen. Target cells were transduced by supplementation of the lentiviral supernatant with 8 μg/mL polybrene. 24 h later, transduced cells were selected by addition of 2 μg/mL puromycin (Bio-connect), a range of 5–10 μg/mL blasticidin (Invivogen) to the medium, or 100 μg/mL hygromycin B (GIBCO) to the medium.

### qPCR

Total RNA was isolated using Trizol reagent (Thermo Fisher Scientific) following the manufacturer’s instructions. Briefly, cells were washed with PBS, and 500 μL Trizol was added for harvesting the cells. After mixing with chloroform (Sigma Aldrich) and centrifuge, the aqueous phase was transferred to a new tube and mixed with 2-Propanol (Sigma Aldrich) for RNA precipitation by centrifuging at 4 °C for 30 min. RNA pellet was washed with 70% ethanol and finally dissolved in RNase-free water (Life Technologies).

Reverse transcription was performed with Tetro Reverse Transcriptase kit (Bioline) according to the manufacturer’s instructions using 1 μg of total RNA per reaction. qPCR products were prepared using a SensiFAST SYBR No-ROX kit (Bioline) according to the instructions on a QuantStudio 5 machine (Applied Biosystems). Data were analyzed by use of the ^ΔΔ^Ct method.

### Incucyte proliferation assay

A total of 2000 cells were seeded per well in 96-well plates (Greiner) with 100 µl of medium. After 24 h, cells were washed with PBS, and the medium was replaced with either complete RPMI or histidine-depleted RPMI at varying levels (50%, 70%, 90%, or full depletion). In certain conditions, histidine was fully removed and then re-supplemented after 48 h. Cells were monitored for up to 196 h. Phase-contrast images were acquired every 8 h using the Incucyte ZOOM system (Essen Bioscience, Ann Arbor, MI, USA), with representative time points selected for analysis due to occasional technical exclusions. Cell proliferation was quantified based on confluence measurements. Each proliferation assay was conducted in at least two independent experiments with six replicates per condition, and mean values are reported.

### Western blotting

Straight lysates from cells were made in 6 wells by the addition of 200 μL of 1x Laemmli buffer without 2-mercaptoethanol and bromophenol blue. Samples were boiled, and protein content was assessed by performing BCA protein quantification (Thermofisher). Then, 2-mercaptoethanol and bromophenol blue were added, and the same amount of protein per sample (between 20 and 30 μg) was loaded and run on SDS-PAGE gels and blotted on 22 μm pore size nitrocellulose membranes (Santa Cruz). URM1 staining was performed using rabbit anti-URM1 polyclonal antibody (ProteinTech, 15285-1-AP; 1:1000), ELP3 staining with rabbit anti-ELP3 monoclonal antibody (Cell Signaling, #5728; 1:1000), HA-tag was visualized with HA-tag polyclonal antibody (Invitrogen, #71-5500, 1:1000), V5 staining was performed using a V5 tag monoclonal antibody (Invitrogen, R960-25; 1:1000) and tubulin with anti-tubulin (DM1A, Sigma, 1:10,000). Subsequent staining was performed with the appropriate LICOR secondary antibodies. Visualization was performed by use of an Odyssey infrared scanning device (LI-COR).

### Flow cytometry analyses and O-Propargyl-Puromycin (OPP) measurements

For the FACS-based staining of T7 tags, cells were seeded in 6-well plates and treated the next day by incubating them with mock or histidine-depleted medium. Triplicates were prepared for each condition tested. After treatment, cells were rinsed once with 1 mL of PBS and detached from the plate by incubating them with 300 μL of PBS-0.5 mM EDTA (0.5 M EDTA, pH 8.0, Invitrogen, #AM9260G) for 10–20 min at 37 °C. Cells were then transferred to a V-shaped 96 well-plate on ice and centrifuged for 4 min at 2000 rpm at 4 °C. The supernatant was discarded, and blocking was performed by resuspending the cells in 150 μL of PBS0.5% BSA and by centrifuging for 4 min at 2000 rpm at 4 °C. Next, cells were resuspended in 40 μL of PBS-0.1% BSA containing the anti-T7 tag primary antibody (Thermo Fisher Scientific, #MA5-41081, 1:200) and incubated on ice for 2 h. After the incubation, cells were centrifuged for 4 min at 2000 rpm at 4 °C, the supernatant was discarded, and two washes with 200 μL of PBS-0.1% BSA were performed. Cells were then resuspended in 50 μL of PBS-0.1% BSA containing the APC-conjugated anti-mouse IgG secondary antibody (Invitrogen, 17-4010-82, 1:500) and incubated in the dark on ice for 1 h. After the incubation, cells were centrifuged for 4 min at 2000 rpm at 4 °C. The supernatant was discarded, and two washes with 200 μL of PBS-0.1% BSA were performed. Finally, cells were resuspended in 200 μL of PBS-0.1% BSA containing DAPI (1:5000) and the samples were analyzed using an Attune NxT machine (Thermo Fisher Scientific). The APC fluorochrome was detected through the 637 nm laser (RL-1, filter 670/14) and DAPI through the 405 nm laser (VL-1, filter 440/50). The data were then analyzed using tFlowJo V10 software (FlowJo).

For OPP measurements, cells transduced with NT control or oligonucleotides targeting URM1 (Supplementary Table S1) were seeded. The next day, cycloheximide (CHX) was added as a control for 10 min, as it should stop protein synthesis. Subsequently, 10 μM of OPP (Life Technologies) was added, and incorporation was allowed for 60 min at 37 °C. Subsequently, the cells were collected by trypsinization and centrifugation and fixed in 70% ethanol overnight at 4 °C. Next, the cells were washed with PBS, permeabilized with 0.1% Triton X-100 (Sigma), and blocked with 3% bovine serum albumin (Sigma) in PBS. Thereafter, the click-it reaction was performed using click-it reagents and picol azide AF647 (all from Thermo Fisher Scientific). The cells were analyzed on an Attune NxT machine (Thermo Fisher Scientific). The data were analyzed using FlowJo V10 software (FlowJo).

### Growth competition assay

At time zero, knockout PANC-1 pancreatic cancer cells for URM1 (KO1 and KO2) were transduced with GFP or mCherry plasmids. The URM1 KO1 and KO2 cells were co-cultured at a 1:1 ratio with NT PANC-1 cells in 6-well plates starting at day 0. Six replicates per condition were used for each treatment group, which included a mock control medium (Ctrl) or a histidine-depleted medium (−H) group, for 5 days, followed by 2 days of recovery in mock medium treatment for both groups. Flow cytometry analysis was conducted at days 0, 4, and 7 using an Attune NxT flow cytometer (Thermo Fisher Scientific). The resulting data were analyzed with FlowJo V10 software (FlowJo).

### Proteomics sample preparation

In IP experiments, beads were heated for 7 min at 95 °C in 1x S-Trap Lysis buffer (5% SDS, 50 mM TEAB pH 8.5), after which proteins were reduced with 20 mM DTT (20 min at 55 °C), alkylated with 40 mM iodoacetamide (30 min at RT in the dark) and digested overnight at 37 °C with trypsin (Sigma-Aldrich; 2 µg per sample) on S-Trap Micro spin columns following the manufacturer’s instructions (ProtiFi, NY, USA). Peptides were eluted, vacuum dried, and stored at 80°C until LC-MS/MS analysis. For single-shot proteomes to be analyzed with data-independent acquisition (DIA), cell pellets were lysed and sonicated in 1x S-Trap lysis buffer, after which aliquots comprising 30 µg of protein were processed as described above, using 3 µg trypsin per sample and STrap Micro spin columns. For 2D-LC-MS/MS, cell pellets were processed in the same way, after which 200 µg aliquots of protein were digested with 20 µg trypsin using S-Trap Mini spin columns. Peptides were eluted, vacuum dried, and stored at −80 °C until further processing. When 9- or 10-plex TMT labeling was employed for 2D-LC-MS/MS, 75 µg peptide aliquots of each sample to be included in a sample pool mix were labeled with 375 µg of TMT-126, -127N/C, -128N/C, -129N/C, -130N/C, or 131 isobaric tag (Thermo Scientific) according to the manufacturer’s instructions. After checking labeling efficiencies of individual samples, labeled digests to be compared were mixed in a 1:1 fashion to create a pool of 9 or 10 samples, dried down in a Speedvac, and stored at −80 °C until further processing. 100 µg (individual, non-labeled samples) or 300 µg (TMT pools) digest were subjected to basic reversed-phase (HpH-RP) high-performance liquid chromatography for offline peptide fractionation as described previously [4], with a slight modification in gradient steepness to account for increased peptide hydrophobicity stemming from TMT labeling. For 2D-LC-MS/MS on breast tumors harvested from mice, tissues were lysed, and proteins were reduced and alkylated in heated guanidine lysis buffer using a probe sonicator as described previously [4]. Proteins were digested, and 150 µg digest of each tumor sample was subjected to HpH-RP as described above. For all 2D-LC-MS/MS samples, collected fractions were concatenated to 12 fractions (DIA LC-MS/MS analyses) or 24 fractions (DDA and TMT LC-MS/MS analyses), vacuum dried, and aliquots comprising ~1 µg peptides of each concatenated fraction were analyzed by nanoLC-MS/MS. Immunopeptidomics sample preparation was performed as described previously [36].

### Proteomics mass spectrometry

For IP experiments, peptides were analyzed by data-dependent acquisition (DDA) LC-MS/MS on an Orbitrap Exploris 480 Mass spectrometer connected to an Evosep One nano-LC system (Evosep Biotechnology, Odense, Denmark). Prior to LC separation with the Evosep One, peptides were reconstituted in 0.1% formic acid, and 10% of the sample was loaded on Evotip PureTM tips (Evosep). Peptides were eluted directly-on-column and separated using the pre-programmed “Extended Method” (88 min gradient) on an EV1137 nano-LC column (heated to 40 °C) with an EV1086 emitter (both Evosep). Nanospray was achieved using the Easy-Spray NG Ion Source (Thermo Scientific). The Exploris 480 was operated in DDA Cycle Time mode with 1 s duty cycle and full MS scans being collected in the Orbitrap analyzer at 60,000 resolutions at m/z 200 over a 375–1500 m/z range. The default charge state was set to 2+, the AGC target was set to “standard” for both MS1 and ddMS2, and for both scan types, the maximum injection time mode was set to “auto.” Monoisotopic peak determination was set to “peptide” and dynamic exclusion was set to 20 s. For ddMS2, a normalized HCD collision energy of 30% was applied to precursors with a 2+ to 6+ charge state meeting a 5e4 intensity threshold filter criterion. Precursors were isolated in the Quadrupole analyzer with a 1.2 m/z isolation window, and MS2 spectra were acquired at 15,000 resolutions in the Orbitrap.

For proteomes, single-shot DIA analyses were performed on the Orbitrap Astral mass spectrometer (Thermo Scientific) connected to a Vanquish Neo nano-LC system (Thermo Scientific) in a 30 samples-per day (30SPD) LC-MS method. The Vanquish Neo was operated in the trap-and-elute mode, and peptides were loaded onto a Pepmap 100 C18 5 µm trap column (300 µm × 5 mm, Thermo Scientific), before separation on the analytical column (AUR3-25075C18-TS, 1.7 µm/75 µm × 25 cm, IonOpticks AU) mounted into an Easyspray ion source (Thermo Scientific). The column was heated at 50 °C, and the flow rate was set to 0.5 µL/min at the start of the method to minimize delay time. Solvent A was 0.1% formic acid/water, and solvent B was 0.1% formic acid/80% acetonitrile. Peptides were eluted at a flow rate of 0.4 µL/min in a 36-min effective gradient, containing a non-linear increase from 8% to 45% solvent B, followed by a 0.4-min ramp to 99% solvent B and 5.4-min wash at 0.5 µL/min flow rate at the end. The column was finally equilibrated using the “fast equilibration” script in combined control mode with a 1450 bar pressure limit. The Orbitrap Astral was run in DIA mode, with full MS scans being collected in the Orbitrap analyzer at 240,000 resolutions at m/z 200 over a 380–980 m/z range. Default charge state was 2+, the normalized AGC target was set to 500% (equivalent to 5e6 charges), and the maximum injection time was 5 ms. For DIA MS2, a normalized HCD collision energy of 25% was applied to a 380–980 m/z precursor range using non-overlapping isolation windows of 2Th, with window placement optimization turned on. Scans were acquired in the Astral analyzer over a 100–1000 m/z range, with the normalized AGC target set to 500% (equivalent to 5e4 charges) and a maximum injection time of 3 ms. DIA analyses of non-labeled HpH-RP fractions were performed using the same instrumental setup and settings, but with a shorter (70SPD) LC gradient.

2D-LC-MS/MS experiments analyzed with DDA were performed on either the Orbitrap Fusion Tribrid (Thermo Scientific) as described previously [2], or on the Orbitrap Exploris 480. Separation of TMT-labeled peptides analyzed on the Exploris 480 was achieved using the Evosep One nano-LC setup described above. The mass spectrometer was operated in DDA Cycle Time mode with 2 s duty cycle and full MS scans being collected in the Orbitrap analyzer with 60,000 resolutions at m/z 200 over a 375–1500 m/z range. Default charge state was set to 2+; the AGC target was set to “standard” for both MS1 and ddMS2, and for both scan types, the maximum injection time mode was set to “auto.” Exclusion duration was set to 20 s. For ddMS2, a normalized HCD collision energy of 36% was applied to precursors with a 2+ to 6+ charge state meeting a 5e4 intensity threshold filter. The fixed first mass was set to m/z 120 because of TMT. Precursors were isolated in the Quadrupole analyzer with a 1.2 m/z isolation window, and MS2 spectra were acquired at 45,000 resolutions in the Orbitrap. Immunopeptidomics LC-MS/MS was performed as described previously [36].

### *MCPT5-cre; Rosa26-DTA* strain and tumor transplantation

The *MCPT5-cre; Rosa26-DTA* transgenic mouse line was kindly provided by Prof. Axel Roers (Institute for Immunology, University Hospital Heidelberg) on a C57BL/6 genetic background. These mice were backcrossed by speed congenics into the FVB/N genetic background. *MCPT5-cre; Rosa26-DTA* mice heterozygous for *MCPT-cre* and heterozygous for *Rosa26-DTA (*het;het mice) are mast cell-deficient, while littermate *MCPT5-cre;Rosa26-DTA* (wt;het) mice are mast cell-proficient controls. Mammary tumor pieces from the transgenic *Keratin14(K14)-cre; Cdh1*^*F/F*^*;Trp53*^*F/F*^ (KEP) mouse model previously described [37] were ortotopically transplanted into female recipient 12–16-week-old wt;het and het;het *MCPT5-cre;Rosa-DTA* mice as previously described [38]. Mammary tumor outgrowth was monitored twice weekly by palpation and caliper measurements. Mice were kept in individually ventilated cages at the animal laboratory facility of the Netherlands Cancer Institute under specific pathogen-free conditions. Food and water were provided ad libitum. All animal experiments were approved by the Netherlands Cancer Institute Animal Ethics Committee (license numbers AVD3010020172688 and AVD30100202215835), and performed in accordance with institutional, national, and European guidelines for Animal Care and Use.

### Histochemistry

Mammary tumors from *MCPT5-cre;Rosa-DTA* wt;het and het;het mice were fixed in 10% neutral buffered formalin, embedded in paraffin, and sectioned at 4 µm. Toluidine blue staining with methyl green counterstain was performed by the Experimental Animal Pathology facility of the NKI. Slides were scanned with the panoramic P1000 slide scanner.

### Mast cells co-culture

*HMC-1 suspension cells* were cultured in RPMI1640 medium (Sigma) supplemented with 1% Glutamax (Gibco), 20 mM HEPES (Gibco), 10% heat inactivated FCS, 50 uM β-mercapto-ethanol (Sigma), and 1% penicillin/streptomycin (Gibco). Medium is refreshed 2–3 times a week when cell density reached 1–1.5 × 10^6^/ml. Cells are re-cultured at a density of 0.25 × 10^6^ cells/ml.

*Prior to co-culture* 1 × 10^4^ HT29 cells were seeded in a 96-well plate in complete DMEM medium. After 24 h 2 × 10^4^ HMC-1 in complete RPMI1640 medium were added and co-cultured with the HT-29 cells for 4 or 7 days. After 4 or 7 days, the medium was collected from the 96-well plate, centrifuged (7 min, 350 g). The collected supernatant (SN) was snap-frozen and stored at −80 °C for use in metabolomics experiments.

For the proteomics experiment, 1 × 10^6^ HT-29 cells were seeded in a 100 mm dish in complete DMEM medium. After 24 h, 2 × 10^6^  HMC-1 in complete RPMI1640 medium were added and co-cultured with the HT29 cells for 7 days. After 7 days, the cells were separated, centrifuged (7 min, 350 g), and washed in PBS. The collected pellet was snap-frozen and stored at −80 °C for use in the proteomics experiments.

### Metabolomics

10 µl of the supernatant from the mast cells co-culture or 10 µl of aqueous calibration samples (MSK-CAA-US-1; Cambridge Isotope Laboratories, Tewksbury, MA, USA) were mixed with 10 µl of internal standard mix (stable isotope labeled amino acid mix (MSK-CAA-1 at 0.25 mM fortified with histamine-d4)). Next, samples were vortex-mixed with 150 µl of ice-cold acetonitrile:formic acid (100:1). The tubes were centrifuged, and 100 µl of supernatant were transferred to a 96-well plate for liquid-chromatography mass spectrometry (LC-MS) analysis. Separation was achieved using an InfinityLab Poroshell 120 HILIC-OH5, 2.1 mm × 100 mm (Agilent) kept at 50 °C and a mobile phase delivered at 0.8 ml/min using linear gradient from 90 to 60% acetonitrile in ammonium formate buffer pH3. The API4000 MS detector (Sciex) was used in MRM mode. Data analysis was done using Analyst 1.7.2.

### RNA sequencing

HT29 cells were treated for 48 h with either control medium, histidine-depleted medium, or tryptophan-depleted medium. Cells were subsequently harvested in RLT buffer (Qiagen). Total RNA was isolated using the RNeasy Mini Kit (74106, Qiagen), including an on column DNase digestion (79254, Qiagen), according to the manufacturer’s instructions. Quality and quantity of the total RNA was assessed by the 2100 Bioanalyzer using “Agilent RNA 6000 Nano” (G2938-90034, Agilent Technologies). Total RNA samples having RIN > 8 were subjected to library generation using the TruSeq stranded mRNA library preparation kit, according to the manufacturer’s instructions (Document # 1000000040498 v00, Illumina) with incorporation of xGen UDI-UMI adapters (Integrated DNA Technologies, Inc., Coralville). The stranded mRNA libraries were analyzed on a 2100 Bioanalyzer instrument following the manufacturer’s protocol “Agilent DNA 7500 kit” (G2938-90025, Agilent Technologies), diluted to 10 nM and pooled equimolar into multiplex sequencing pools for paired-end sequencing on the NovaSeq 6000 Illumina sequencing instrument. Paired-end sequencing was performed using 150 cycles for Read 1, 19 cycles for Read i7, 10 cycles for Read i5 and 150 cycles for Read 2, using the NovaSeq6000 Reagent Kit v1.5 (300 cycles) (20028400, Illumina). On average, 40 M paired reads per sample were generated. Fastq files were aligned using STAR (STAR-2.7.10a) to the human GRCh38 genome. Aligned reads on exons of protein-coding genes were then counted using htseq-count (version 2.0.5) and gencode 38 reference annotation. DESeq2 was used for normalization and differential expression analysis.

### Statistics and general methods

For all experiments, a sample size was chosen that allowed adequate power of statistical testing. The number of samples and proteins used in each comparison is stated in the results. To ensure the correct application of statistical tests variatian in groups was determined using standard deviations. All figures include error bars if appropriate, and IQR ranges and medians are included in boxplots. Most P values, unless otherwise stated, are calculated by Wilcoxon t-test, which is more robust in cases of deviations from normal distribution.

### Analysis of IP/MS data

#### Data generation

At the end of each experiment intended for HA-tag or V5-tag pulldown, cells were treated with 10 μM MG-132 for 4 h and subsequently collected by trypsinization and centrifugation. Next, cells were lysed in 300 μL ELB lysis buffer (50 mM HEPES, 125 mM NaCl, 0.5% (v/v) Tween-20, and 0.1% (v/v) Nonidet P40 Substitute) and snap-frozen in liquid nitrogen. For the V5-IP/MS, samples were then thawed on ice and incubated overnight at 4 °C with 3 μL of anti-V5 antibody. Next, pull-down was achieved by mixing the lysate with “Dynabeads™” ProteinG (Invitrogen) and by incubating at room temperature for 30 min. Samples were then washed three times with 400 μL of PBS-0.01% Tween-20 and resuspended in 100 μL of PBS-0.01% Tween-20. After removing the supernatant, the dry beads were stored at −80 °C until the subsequent mass spectrometry analysis (as previously described in [2]).

For the HA-IP/MS, the samples were thawed on ice, and in order to assess IP efficiency, 20 μL of the sample was collected before the IP. Anti-TMT-Magnetic Beads (Sigma) were washed twice with the ELB lysis buffer (see above). Then the samples were incubated overnight at 4 °C with 3 μL of anti-HA Magnetic Beads. To assess pull-down efficiency, 20 μL of supernatant was collected after the HA-pull-down. Samples were then washed three times with 400 μL of PBS-0.01% Tween-20 and resuspended in 100 μL of PBS-0.01% Tween-20. After removing the supernatant, the dry beads were stored at −80°C until the subsequent mass spectrometry analysis (as previously described in [2]). Samples collected before and after pull-down were run for Western blot analyses.

#### Generation of search database T7 IP/MS

At every histidine position in the T7 reporter sequence, a minus-1 and plus-1 frameshift was introduced in silico. Further, every histidine in the T7 reporter sequence was separately exchanged for every other amino acid, such that for every separate histidine, a substituted peptide was obtained. The WT sequence, as well as the frameshift and substituted sequences, were subjected to in-silico trypsinization, and all peptides with a length between 5 and 55 were kept.

#### Generation of search database arginine IP/MS

At the only histidine in this reporter, a minus-1 and a plus-1 frameshift were introduced in silico. Histidine in the arginine IP reporter sequence was also exchanged for glutamine. The WT sequence, as well as the frameshift and substituted sequences, were subjected to in silico trypsinization, and all peptides with a length between 5 and 55 were kept.

#### Proteomics analysis of IP/MS

IP/MS data were analyzed with MaxQuant (version 2.0.1.0) against custom-made libraries. Peptide false discovery rate (FDR) set at 0.01. Further parameters of the search are deposited in the PRIDE database and supplementary Table S2.

### Proteomics dataset creation for TMT, DDA, and DIA

An initial database included all instances of histidine amino acids in the proteome (UniPROT), changed to all other amino acids except Arginine and Lysine. For later library creation, gencode (version 44, GRCh38.p14) transcripts were obtained, and the two histidine codons CAC and CAT were individually replaced by Glutamine codons. The WT sequences were retained in the library.

Subsequently, mRNA coding sequences of transcripts were in-silico translated into protein sequences.

Tryptic peptides were created, and only peptides ranging from 5 to 55 amino acids in length were kept. UniPROT (UP000005640) amino acid sequences were further added as controls.

### Proteomics dataset creation for DIA analysis in mouse

The library creation follows basic steps described above for human libraries. In short, gencode transcripts (version 37, gencode. vM37) were obtained, and the two histidine codons CAC and CAT were individually replaced by Glutamine codons. The WT sequences were retained in the library.

Subsequently, mRNA coding sequences of transcripts were in-silico translated into protein sequences. Tryptic peptides were created, and only peptides ranging from 5 to 55 amino acids in length were kept. Mouse-specific UniPROT (UP000000589) amino acid sequences were further added as controls.

### Analysis of large-scale proteomics data of human cancer and TMT data

Mass spectrometry-derived processed spectra (mzML) files for all independent cancer types were downloaded from the Proteomics Data Center [18]. In an initial run, a database of protein sequences was created that includes the human proteome with all instances of histidine amino acids in the proteome changed to all other amino acids except Arginine and Lysine, and the canonical human proteome (UniPROT). The substitutant tryptic peptides are kept with a length of >5 and <50 amino acids. Later analyses of CPTAC datasets were performed with a custom library designed as explained in the section below. Regarding the analysis, MSFragger searches the mzML spectral files against the custom database for peptide detection with the following parameters: Precursor mass lower: −20 ppm, Precursor mass upper: 20 ppm, precursor mass tolerance: 20 ppm, calibrate mass: True, Deisotoping:

True, mass offset: False, isotope error: Standard, digestion: Strictly tryptic (Max. missed cleavage: 2), Variable modifications: 15.99490 M 3, 42.01060 [^ 1, 144.1021 n^ 1, 144.1021 S 1, Min Length: 7, Max Length: 50, digest mass range: 500:5000 Daltons, Max Charge: 2, remove precursor range: −1.5, 1.5, topN peaks: 300, minimum peaks: 15, precursor range: 1:6, add Cysteine: 57.021464, add Lysine: 229.162932, among other basic parameters (Supplementary Table S3)]. Next, PeptideProphet validates detected peptides with the following parameters: accmass: TRUE, decoyprobs: TRUE, expectScore: TRUE, Glycosylation: FALSE, ICAT: FALSE, masswidth: 5, minimum probability after first pass of a peptide: 0.9, minimum number of NTT in a peptide: 2, among other parameters (Supplementary Table S3). Isobaric quantification was then undertaken using the following parameters (bestPSM: TRUE, level: 2, minProb 0.7, ion purity cut-off: 0.5, tolerance: 20 ppm, among other parameters (Supplementary Table S3)). Next, to retain only confident peptides, peptides were filtered using stringent False Discovery Rate (FDR) filtering. The following parameters were used for FDR filtering: FDR < 0.01, peptide Probability: 0.7, among other parameters (Supplementary Table S3). Next, TMT-integrator was used to create integrated reports with isobaric quantification across all samples with the following parameters (retention time normalization: False, minimum peptide probability on top of FDR filtering: 0.9, among other parameters (Supplementary Table S3)). Substitutant peptides were fetched from the reports of TMT Integrator. Using custom scripts, peptides with a log2-transformed intensity score above 0 in a sample were observed as positively detected peptides in that sample.

### Analysis of specific protein impact and correlation in the CPTAC dataset

Survival data and log2-normalized mRNA expression data of CPTAC datasets were obtained from [39]. The number of H>Q peptides per tumor sample was obtained from our own proteomics runs. The data was merged on the tumor sample case ID. Tumor samples were divided in high protein expression or high H>Q number based on being above or below the mean of this dataset. Survival analyses were subsequently performed using the R survival package.

### Data generation from 2D proteomics data

On the first day, cells were seeded in 15 cm dishes at around 70% confluency. The next day, cells were rinsed with PBS and were exposed to the appropriate treatment histidine or tryptophan-free medium. As a control, RPMI 1640 (GIBCO) full media has been used. After 48 h of treatment, 10 μM MG-132 was added directly to the plates, and cells were incubated for 4 h at 37 °C. Then, cells were washed once with PBS and collected by trypsinization and centrifugation. Cell pellets were washed once with PBS, after which they were snap-frozen in liquid nitrogen.

### Analysis of DDA proteomics data

MSFragger was used, which searches the mzML spectral files against the given database for peptide detection with the following parameters. Precursor mass lower: −20 ppm, Precursor mass upper: 20 ppm, precursor mass tolerance: 20 ppm, calibrate mass: True, Deisotoping: True, mass offset: False, isotope error: Standard, digestion: Strictly tryptic (Max. missed cleavage: 2), Variable modifications: 15.99490 M 3, 42.01060 [^ 1, Min Length: 7, Max Length: 50, digest mass range: 500:5000 Daltons, Max Charge: 2, remove precursor range: −1.5, 1.5, topN peaks: 150, minimum peaks: 15, add Cysteine: 57.021464, add Lysine: 229.162932, among other basic parameters (Supplementary Table S4)]. Next, PeptideProphet validates detected peptides with the following parameters: accmass: TRUE, decoyprobs: TRUE, expectScore: TRUE, Glycosylation: FALSE, among other parameters (Supplementary Table S4).

### Analysis of DIA proteomics data

DIA datasets in mzML format were analyzed using diann (version 1.8.1) with the following parameters. diann-1.8.1 --cut “K*,R*,!*P” --dir mzml/ --fasta H2Q_gencode.v44.fasta --fasta-search fixed-mod UniMod:4,57.021464,C --gen-spec-lib --lib “” --matrices --missed-cleavages 1 -metexcision --min-fr-min 200 --max-fr-mhz 1800 --min-pep-len 5 --max-pep-len 50 --min-pr-mz 300 -max-pr-mhz 1800 --min-pr-charge 1 --max-pr-charge 4 --predictor --qvalue 0.01 --relaxed-prot-inf reanalyze --smart-profiling --threads 64 --use-quant --var-mods 5 --var-mod UniMod:1,42.010565,*n -var-mod UniMod:35,15.994915,M----verbose 1.

### H>Q selection

H>Q enriched peptides in TMT datasets were defined as H>Q peptides that have a TMT ratio that is higher than the mean ratio of ctrl peptides in histidine depletion condition. Control peptides are defined as depleted (in H>Q). Within the depleted control, we distinguish between high and low based on expression levels (ratio above 0.1). In DIA and DDA proteomics, H>Q enriched peptides were defined as peptides that are expressed in all histidine depletion replicates and in none of the control replicates. Top control peptides (non-H>Q top) were selected by sorting for highest mean intensity in control replicates and selecting a number equal to the found H>Q peptides (Supplementary Fig. S1A). For samples with URM1 KO and URM1 mutant in PANC-1 and Miapaca2, H>Q were selected, which are expressed in all the replicates. In these samples shared background peptides expressed in all samples were not removed.

### Signature plots

Signature plots were created using custom Python scripts. H>Q peptides found in the mass spectronomy experiments were mapped back to their full-length protein sequence in the previously described custom gencode reference file. Then the substituted H was centered, and a sequence window of 30 nucleotides before and after the Histidine codon was extracted. Peptides that were too close to the start codon or the stop codon to generate a 30-nucleotide window were discarded. For each position in the 60-nucleotide window, the number of A, C, G, and T nucleotide occurrences was counted. A fraction was calculated by dividing these positional numbers by the total number of the corresponding nucleotide in the 60 nt window. Thereby determining how many nucleotides of all existing nucleotides fall on this position. As a baseline, all histidine-containing expressed WT peptides were used that were captured in the same mass spectrometry run. In these peptides, each Histidine was mapped to the canonical gencode transcripts, and then the previously described calculation steps were performed. For the actual plot, the obtained baseline WT fractions per position were subtracted from the H>Q fractions, and the difference in fraction per position was plotted separately per nucleotide. As a control in the signature analysis, peptides of the highest intensity WT peptides (non-H>Q top) were analyzed in the same manner (all Histidine codons in these found peptides centered) to perform a signature analysis with them. *Signature heatmaps*: For a window of 3 nucleotides before and after the Histidine, the calculated fractions per position were extracted. This creates a dataset of the 6nt window (−3 to +3) with the fractions of each of the four nucleotides. Then, for each of the positions in the dataset, the fraction of each nucleotide to the total amount of all nucleotides on this position was calculated (nuc fraction/(Afraction + Cfraction + Gfraction + Tfraction)). These were then again subtracted from the (normal) control, and the difference was plotted in a heatmap.

### Immunopeptidomics

A database for immunopeptidomics was prepared based on the UniProt database UP000005640. All instances of canonical “Histidine” were identified, and a sequence of 10 amino acids upstream and downstream (−10 Upstream- W- +10 Downstream) was extracted. In this sequence window, all histidine residues were substituted with glutamine. This sequence collection was concatenated to the Uniprot database. Database search was performed using Fragpipe v20.0 with its native nonspecific-HLA workflow (preset parameters) to identify immunopeptides. Manifest files listed all RAW files with the corresponding experiment ID. Each replicate was scanned independently. The files were scanned in DDA mode. The parameter file for the search is also deposited in the PRIDE Database. FragPipe scoring metrics were used in order to obtain the retention time, nextscore, hyperscore, and probability. We extracted this information from the individual PSM (Peptide Spectrum Match) files.

### Protein level analysis

The found H>Q peptides were mapped to the canonical protein they originated from based on the WT gencode versions, as described in library creation. This protein was then included in the “H>Q” group. As a control, the proteins with the highest mean intensity in control conditions (non-H>Q top group) were used. For the boxplots, the mean sum intensity of all replicates in a group was calculated and plotted. The *p* value (Wilcoxon t-test) between control conditions and histidine depletion conditions was calculated. Basepair (bp) length and GC content calculations of proteins were performed on the longest canonical isoform transcripts in gencode v44. For length in bp, the length of the annotated UTR5′, coding sequence, and UTR3′ was calculated based on coding sequence start- and end-coordinate annotation in the gencode v44 reference file. GC fraction is calculated as the amount of GC nucleotides over the amount of all nucleotides in the annotated coding sequence (sum(GC)/sum(ACGT)). The number of histidine codons in groups was also calculated based on the longest canonical isoform transcripts in gencode v44. Then, for each transcript, the fraction of histidine codons to the total of all codons in 5′ UTR, CDS, and 3′ UTR was calculated (sum(CAC + CAT)/sum(all codons)).

Histidine codon positions on the longest canonical isoform transcripts in gencode v44 were determined, and the percentile position was calculated based on the transcript length (Histidine position × 100 / (length protein)). Gene Ontology (GO) terms for any protein-level analysis were obtained from DAVID webserver using the BP GO terms category [40].

For proteins from the H>Q group, and proteins with the highest mean intensity in control conditions (non-H>Q top), the number of histidines encoded by a CAC codon that is followed by an A or a C nucleotide was determined based on longest canonical isoform transcripts in gencode v44. Then the fraction of CAC+A against CAC+C was calculated for each protein (CACA/CACC). Per cellline the 20 proteins with highest and lowest CACA/CACC ratio were taken, and a unique “top” and “bottom” (based on high- and low-ratio) protein set were created, which were used for GO term analyses with the DAVID webserver as described previously [40]. Histone protein levels were obtained from protein intensity data, based on UniPROT amino acid sequences included in the library as described in the library creation section.

### H>Q abundance analysis

To determine H>Q abundance, all H>Q peptides are mapped to their canonical protein of origin. Then the total summed number of H>Q peptides per protein is defined as those proteins “H>Q abundance.”

## Supplementary information

Supplementary Material

Supplementary Tables S1–S4

Original Data

## Data Availability

Proteomics data have been deposited to the ProteomeXchange Consortium via the PRIDE [41] partner repository with identifier PXD060514 (Reviewer account details: **Token** oGIZAOPXjYdC or **Username** reviewer_pxd060514@ebi.ac.uk, **Password** Zg3rmBGRmiXi). RNA sequencing data are deposited in NCBI GEO under accession number GSE330343 with reviewer token alelyksoztivbgr.

## Code availability

Computational code used for signature analysis and visualization is publicly available on GitHub https://github.com/pkorner218/substitutant_mRNAmotifsscan. Code for signature analysis is written in Python 3 and was tested under Python 3.12. Package-specific version requirements are pandas ≥ 2.0 and plotline ≥ 0.14. Code for plotting in R was tested in R4.3.3.X, and it requires the heatmap library. All code is freely available under the MIT license.

## References

[1] Wright BW, Yi Z, Weissman JS, Chen J. The dark proteome: translation from noncanonical open reading frames. Trends Cell Biol. 2022;32:243–58. 34844857 10.1016/j.tcb.2021.10.010 PMC8934435

[2] Pataskar A, Champagne J, Nagel R, Kenski J, Laos M, Michaux J, et al. Tryptophan depletion results in tryptophan-to-phenylalanine substitutants. Nature. 2022;603:721–7. 35264796 10.1038/s41586-022-04499-2 PMC8942854

[3] Yang C, Pataskar A, Feng X, Montenegro Navarro J, Paniagua I, Jacobs JJL, et al. Arginine deprivation enriches lung cancer proteomes with cysteine by inducing arginine-to-cysteine substitutants. Mol Cell. 2024;84:1904–16.e7. 38759626 10.1016/j.molcel.2024.04.012 PMC11129317

[4] Bartok O, Pataskar A, Nagel R, Laos M, Goldfarb E, Hayoun D, et al. Anti-tumour immunity induces aberrant peptide presentation in melanoma. Nature. 2021;590:332–7. 33328638 10.1038/s41586-020-03054-1

[5] Pavlova NN, Zhu J, Thompson CB. The hallmarks of cancer metabolism: Still emerging. Cell Metab. 2022;34:355–77. 35123658 10.1016/j.cmet.2022.01.007 PMC8891094

[6] Faubert B, Solmonson A, DeBerardinis RJ. Metabolic reprogramming and cancer progression. Science. 2020;368:eaaw5473. 10.1126/science.aaw5473 PMC7227780 32273439

[7] Zhao J, Jin D, Huang M, Ji J, Xu X, Wang F, et al. Glycolysis in the tumor microenvironment: a driver of cancer progression and a promising therapeutic target. Front Cell Dev Biol. 2024;12:1416472. 38933335 10.3389/fcell.2024.1416472 PMC11199735

[8] Rabinovich S, Adler L, Yizhak K, Sarver A, Silberman A, Agron S, et al. Diversion of aspartate in ASS1-deficient tumours fosters de novo pyrimidine synthesis. Nature. 2015;527:379–83. 26560030 10.1038/nature15529 PMC4655447

[9] Nagel R, Kochavi A, Flem-Karlsen K, Adhikary M, Gal-On Y, Klaoudatou MG, et al. Arginine deprivation of ASS1-deficient cancers drives mistranslation and shared neoepitope production. Cancer Res. 2026. 10.1158/0008-5472.CAN-25-4773 PMC13370116 42095547

[10] Sirniö P, Väyrynen JP, Klintrup K, Mäkelä J, Karhu T, Herzig K-H, et al. Alterations in serum amino-acid profile in the progression of colorectal cancer: associations with systemic inflammation, tumour stage and patient survival. Br J Cancer. 2019;120:238–46. 30563990 10.1038/s41416-018-0357-6 PMC6342921

[11] Kumar N, Rachagani S, Natarajan G, Crook A, Gopal T, Rajamanickam V, et al. Histidine enhances the anticancer effect of gemcitabine against pancreatic cancer via disruption of amino acid homeostasis and oxidant-antioxidant balance. Cancers. 2023;15:2593. 37174059 10.3390/cancers15092593 PMC10177467

[12] Akinlalu A, Flaten Z, Rasuleva K, Mia MS, Bauer A, Elamurugan S, et al. Integrated proteomic profiling identifies amino acids selectively cytotoxic to pancreatic cancer cells. Innovation. 2024;5:100626. 10.1016/j.xinn.2024.100626 PMC11063643 38699777

[13] Mayerle J, Kalthoff H, Reszka R, Kamlage B, Peter E, Schniewind B, et al. Metabolic biomarker signature to differentiate pancreatic ductal adenocarcinoma from chronic pancreatitis. Gut. 2018;67:128–37. 28108468 10.1136/gutjnl-2016-312432 PMC5754849

[14] Mandleywala K, Ulrich S, da Silva-Diz V, Sharma P, Thai C, Kim O, et al. A dietary pan-amino acid dropout screen in vivo reveals a critical role for histidine in T-ALL. bioRxiv [Preprint]. 2025. Available from: 10.64898/2025.12.21.694897.

[15] Li H, Xiao Y, Li Q, Yao J, Yuan X, Zhang Y, et al. The allergy mediator histamine confers resistance to immunotherapy in cancer patients via activation of the macrophage histamine receptor H1. Cancer Cell. 2022;40:36–52.e9. 34822775 10.1016/j.ccell.2021.11.002 PMC8779329

[16] Salmerón C, Tomás Bort E, Sriram K, Javadi-Paydar M, Smitham JE, Pham K, et al. Histamine H1 receptor: a potential therapeutic target for pancreatic ductal adenocarcinoma. J Pharmacol Exp Ther. 2025;392:103573. 40288207 10.1016/j.jpet.2025.103573

[17] Termathe M, Leidel SA. Urm1: a non-canonical UBL. Biomolecules. 2021;11:139. 10.3390/biom11020139 PMC7911844 33499055

[18] Edwards NJ, Oberti M, Thangudu RR, Cai S, McGarvey PB, Jacob S, et al. The CPTAC Data Portal: a resource for cancer proteomics research. J Proteome Res. 2015;14:2707–13. 25873244 10.1021/pr501254j

[19] Huang H, Li Y, Liang J, Finkelman FD. Molecular regulation of histamine synthesis. Front Immunol. 2018;9:1392. 10.3389/fimmu.2018.01392 PMC6019440 29973935

[20] Parker J, Pollard JW, Friesen JD, Stanners CP. Stuttering: high-level mistranslation in animal and bacterial cells. Proc Natl Acad Sci USA 1978;75:1091–5. 349556 10.1073/pnas.75.3.1091 PMC411414

[21] Laxman S, Sutter BM, Wu X, Kumar S, Guo X, Trudgian DC, et al. Sulfur amino acids regulate translational capacity and metabolic homeostasis through modulation of tRNA thiolation. Cell. 2013;154:416–29. 23870129 10.1016/j.cell.2013.06.043 PMC3757545

[22] Van der Veen AG, Schorpp K, Schlieker C, Buti L, Damon JR, Spooner E, et al. Role of the ubiquitin-like protein Urm1 as a noncanonical lysine-directed protein modifier. Proc Natl Acad Sci USA 2011;108:1763–70. 21209336 10.1073/pnas.1014402108 PMC3033243

[23] Khoshnood B, Dacklin I, Grabbe C. Urm1: an essential regulator of JNK signaling and oxidative stress in Drosophila melanogaster. Cell Mol Life Sci. 2016;73:1939–54. 26715182 10.1007/s00018-015-2121-x PMC11108535

[24] Rapino F, Delaunay S, Zhou Z, Chariot A, Close P. tRNA modification: Is cancer having a wobble? Trends Cancer. 2017;3:249–52. 28718436 10.1016/j.trecan.2017.02.004

[25] Sullivan MR, Danai LV, Lewis CA, Chan SH, Gui DY, Kunchok T, et al. Quantification of microenvironmental metabolites in murine cancers reveals determinants of tumor nutrient availability. eLife. 2019;8:e44235. 10.7554/eLife.44235 PMC6510537 30990168

[26] Amobi A, Qian F, Lugade AA, Odunsi K. Tryptophan catabolism and cancer immunotherapy targeting IDO mediated immune suppression. Adv Exp Med Biol. 2017;1036:129–44. 29275469 10.1007/978-3-319-67577-0_9

[27] Chen CL, Hsu SC, Ann DK, Yen Y, Kung HJ. Arginine signaling and cancer metabolism. Cancers. 2021;13:3541. 10.3390/cancers13143541 PMC8306961 34298755

[28] Agris PF, Vendeix FA, Graham WD. tRNA’s wobble decoding of the genome: 40 years of modification. J Mol Biol. 2007;366:1–13. 17187822 10.1016/j.jmb.2006.11.046

[29] Huang B, Johansson MJ, Byström AS. An early step in wobble uridine tRNA modification requires the Elongator complex. RNA. 2005;11:424–36. 15769872 10.1261/rna.7247705 PMC1370732

[30] Tavares JF, Davis NK, Poim A, Reis A, Kellner S, Sousa I, et al. tRNA-modifying enzyme mutations induce codon-specific mistranslation and protein aggregation in yeast. RNA Biol. 2021;18:563–75. 32893724 10.1080/15476286.2020.1819671 PMC7971265

[31] Nedialkova DD, Leidel SA. Optimization of codon translation rates via tRNA modifications maintains proteome integrity. Cell. 2015;161:1606–18. 26052047 10.1016/j.cell.2015.05.022 PMC4503807

[32] Costa-Mattioli M, Walter P. The integrated stress response: from mechanism to disease. Science. 2020;368:eaat5314. 32327570 10.1126/science.aat5314 PMC8997189

[33] Jiang C, Tan X, Jin J, Wang P. The molecular basis of amino acids sensing. Adv Sci. 2025;12:2501889. 10.1002/advs.202501889 PMC12244511 40411419

[34] Dong N, Ma HX, Liu XQ, Li D, Liu LH, Shi Q, et al. Histidine re-sensitizes pediatric acute lymphoblastic leukemia to 6-mercaptopurine through tetrahydrofolate consumption and SIRT5-mediated desuccinylation. Cell Death Dis. 2024;15:216. 38485947 10.1038/s41419-024-06599-5 PMC10940622

[35] Frezza C. Histidine metabolism boosts cancer therapy. Nature. 2018;559:484–5. 30030511 10.1038/d41586-018-05573-4 PMC6445353

[36] Champagne J, Nielsen MM, Feng X, Montenegro Navarro J, Pataskar A, Voogd R, et al. Adoptive T cell therapy targeting an inducible and broadly shared product of aberrant mRNA translation. Immunity. 2025;58:247–62.e9. 39755122 10.1016/j.immuni.2024.12.004

[37] Derksen PW, Liu X, Saridin F, van der Gulden H, Zevenhoven J, Evers B, et al. Somatic inactivation of E-cadherin and p53 in mice leads to metastatic lobular mammary carcinoma through induction of anoikis resistance and angiogenesis. Cancer Cell. 2006;10:437–49. 17097565 10.1016/j.ccr.2006.09.013

[38] Blomberg OS, Spagnuolo L, Garner H, Voorwerk L, Isaeva OI, van Dyk E, et al. IL-5-producing CD4+ T cells and eosinophils cooperate to enhance response to immune checkpoint blockade in breast cancer. Cancer Cell. 2023;41:106–23.e10. 36525971 10.1016/j.ccell.2022.11.014

[39] Vasaikar SV, Straub P, Wang J, Zhang B. LinkedOmics: analyzing multi-omics data within and across 32 cancer types. Nucleic Acids Res. 2018;46:D956–63. 29136207 10.1093/nar/gkx1090 PMC5753188

[40] Sherman BT, Hao M, Qiu J, Jiao X, Baseler MW, Lane HC, et al. DAVID: a web server for functional enrichment analysis and functional annotation of gene lists (2021 update). Nucleic Acids Res. 2022;50:W216–21. 35325185 10.1093/nar/gkac194 PMC9252805

[41] Perez-Riverol Y, Bai J, Bandla C, García-Seisdedos D, Hewapathirana S, Kamatchinathan S, et al. The PRIDE database resources in 2022: a hub for mass spectrometry-based proteomics evidences. Nucleic Acids Res. 2021;50:D543–52. 10.1093/nar/gkab1038 PMC8728295 34723319

