# Supplementary material for: Histidine to glutamine substitutants, a regulated process that impacts cell survival and suggests histidine shortage in cancer
Source: Cell Death Discov. 2026 Jun 27;12:368. doi: 10.1038/s41420-026-03225-5 (PMC13572455; doi:10.1038/s41420-026-03225-5)

S3B_URM1_MW+ Lane 1-4

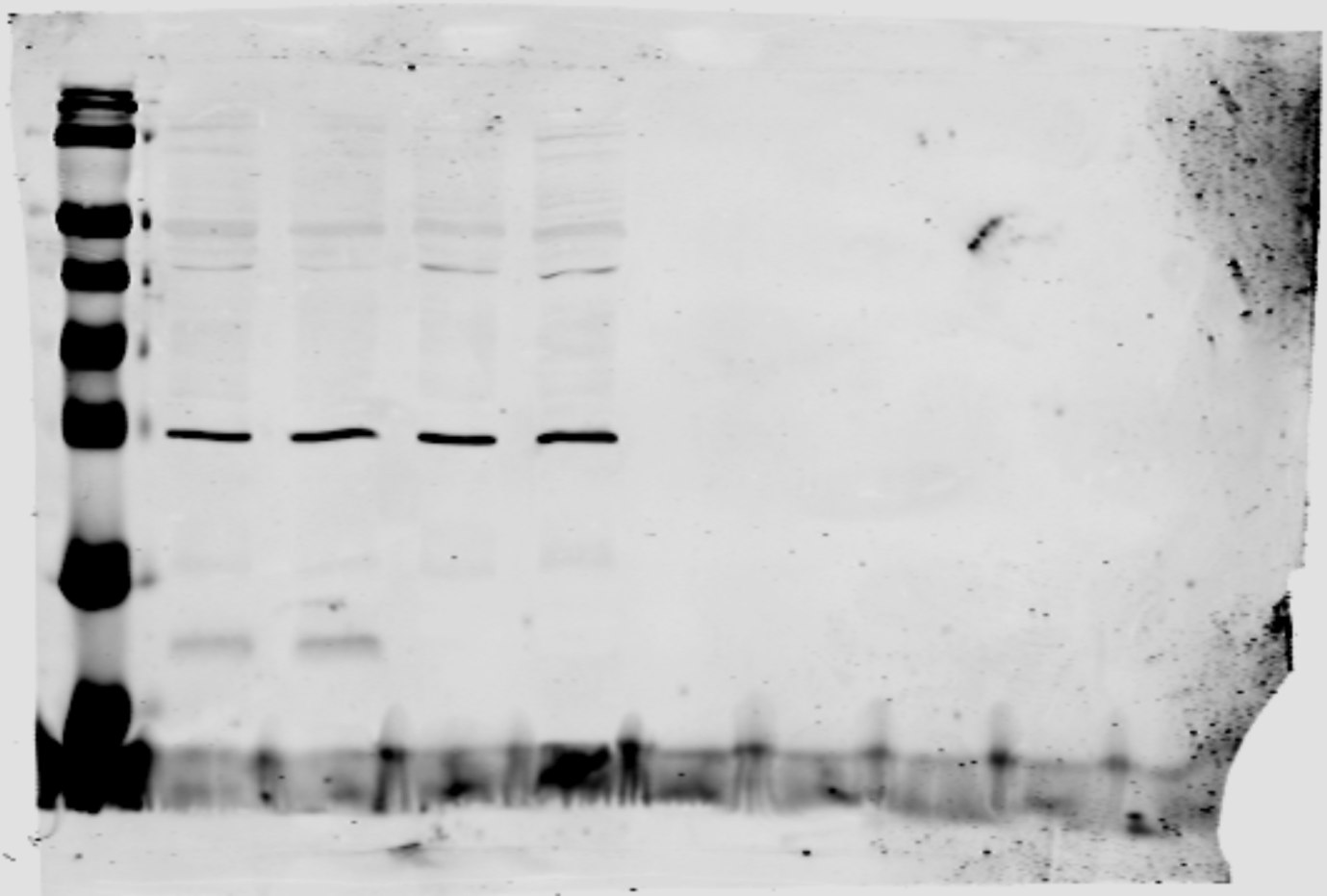

S3B_Tubulin_ Lane 1-4

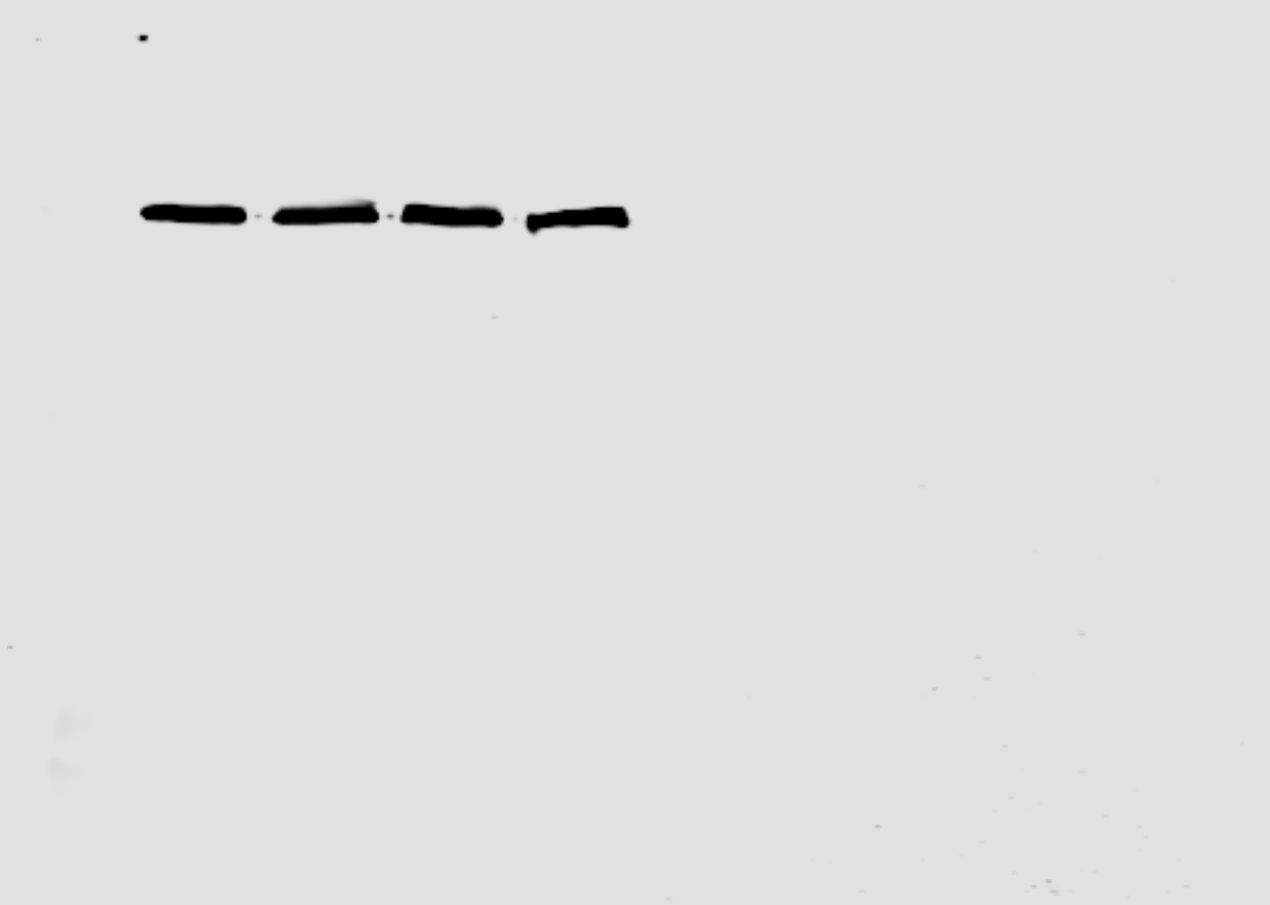

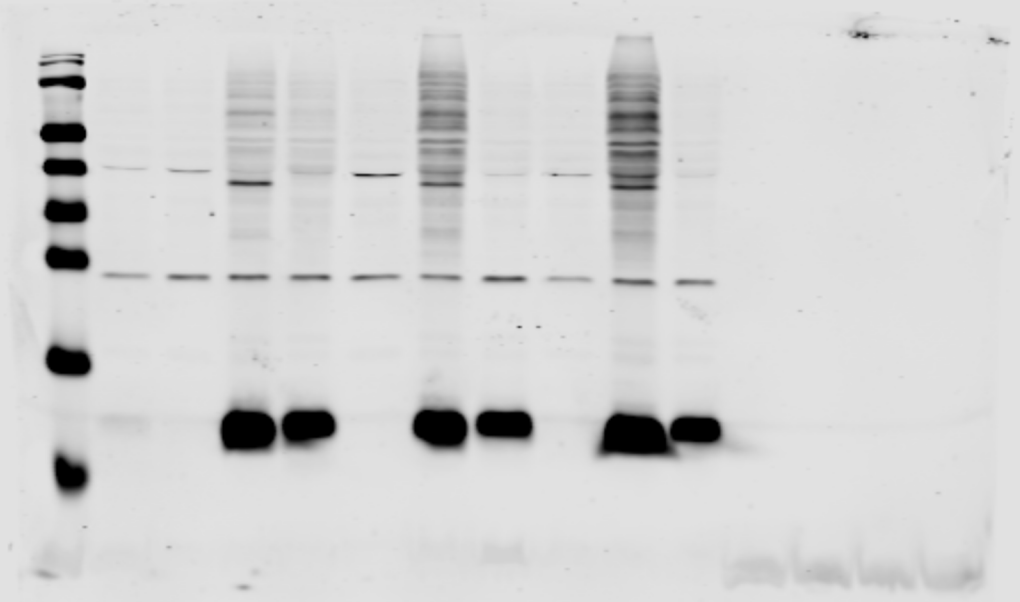

S3C (Below)- URM1 staining _MW+ Lane 1-5

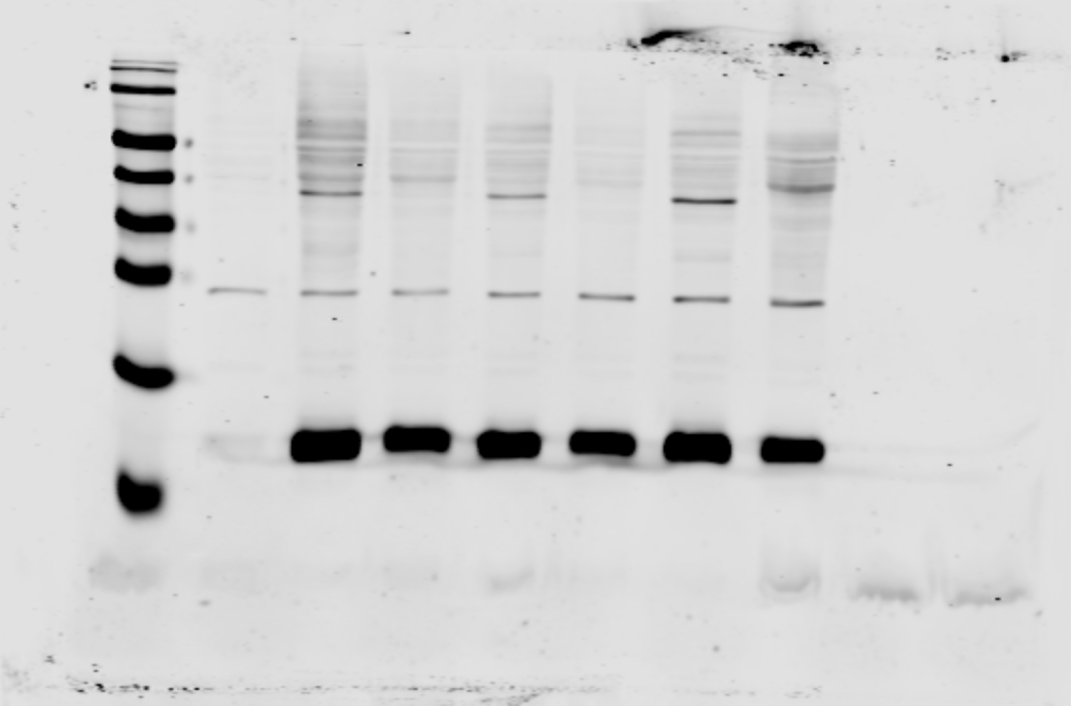

S3C (Below)- Tubulin staining _MW+ Lane 1-5

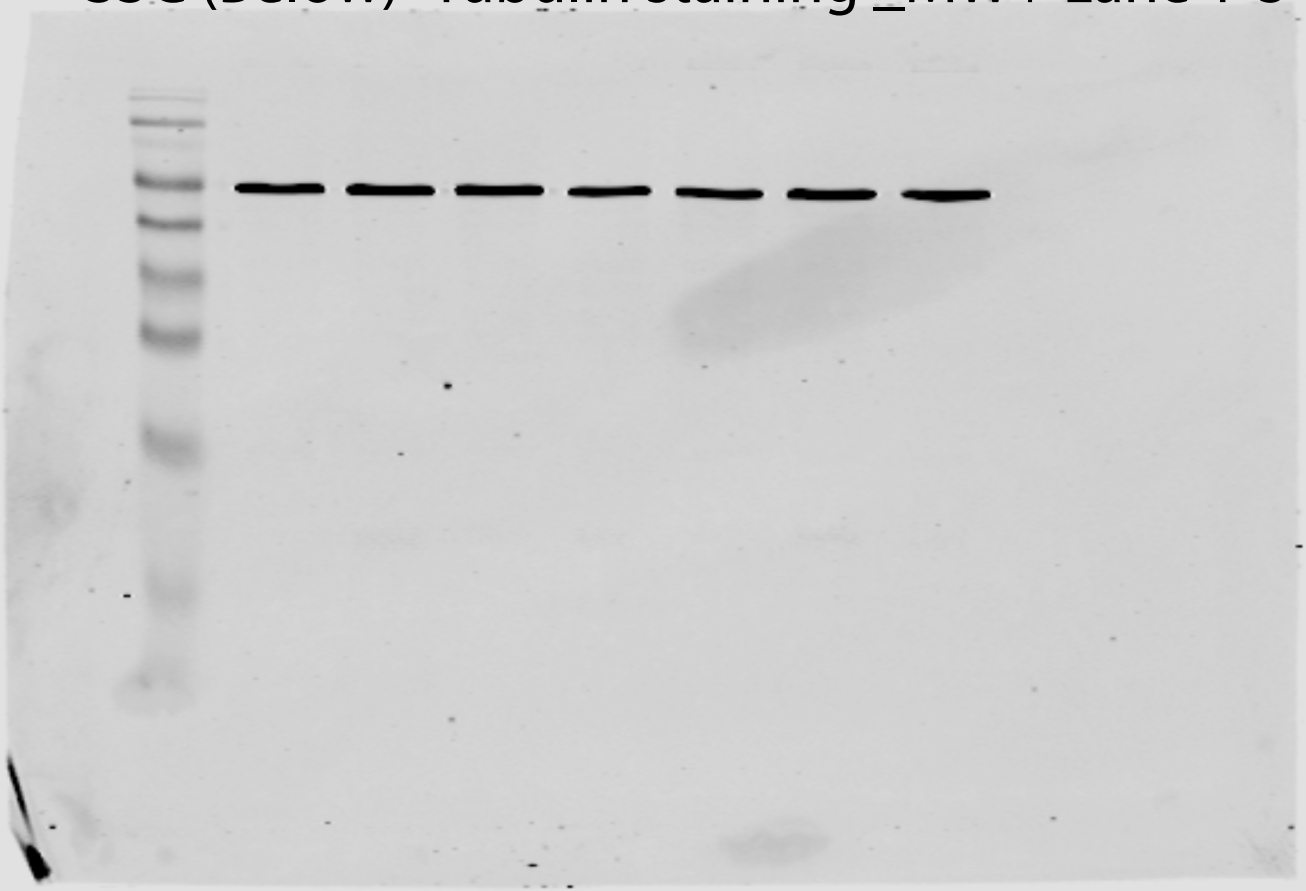

S4A_URM1_ MW+ Lane 1-4

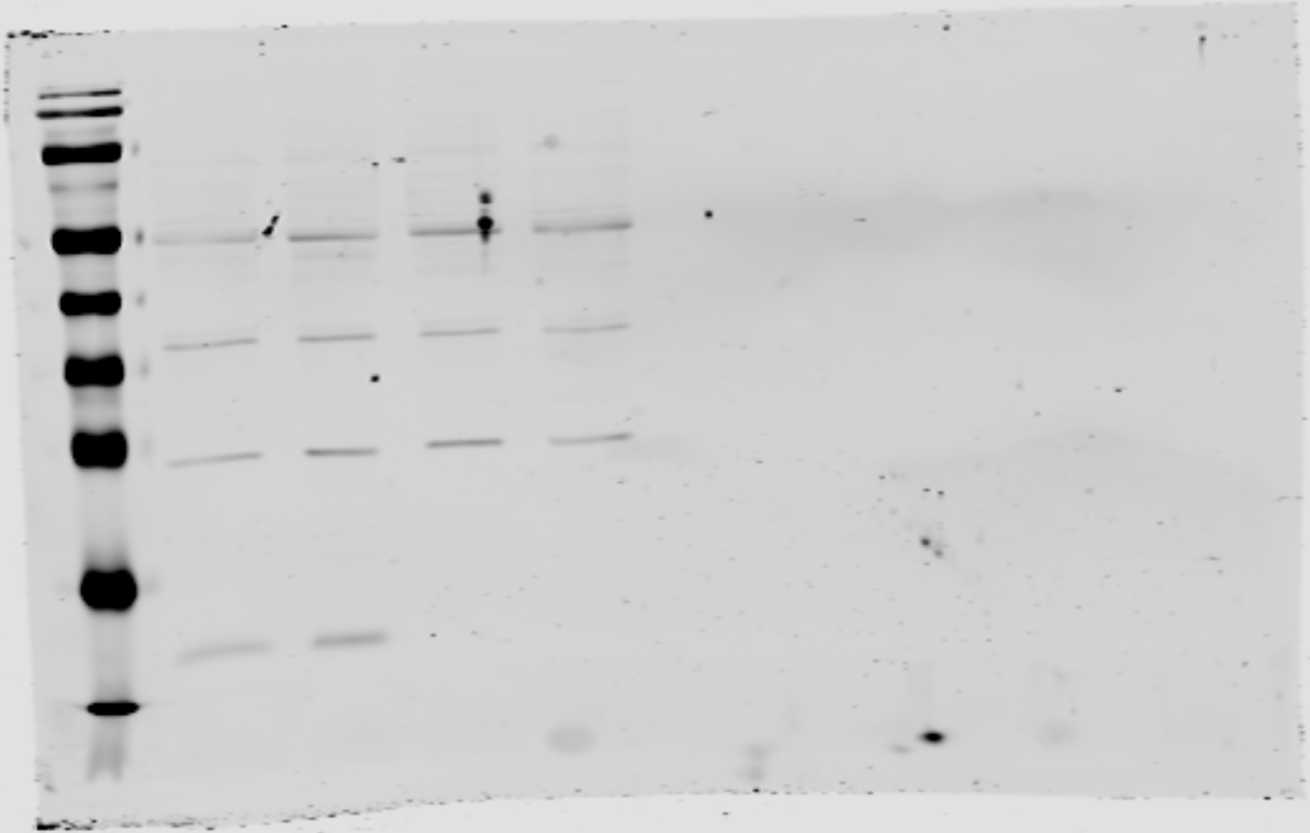

S4A_Tubulin_ Lane 1-4

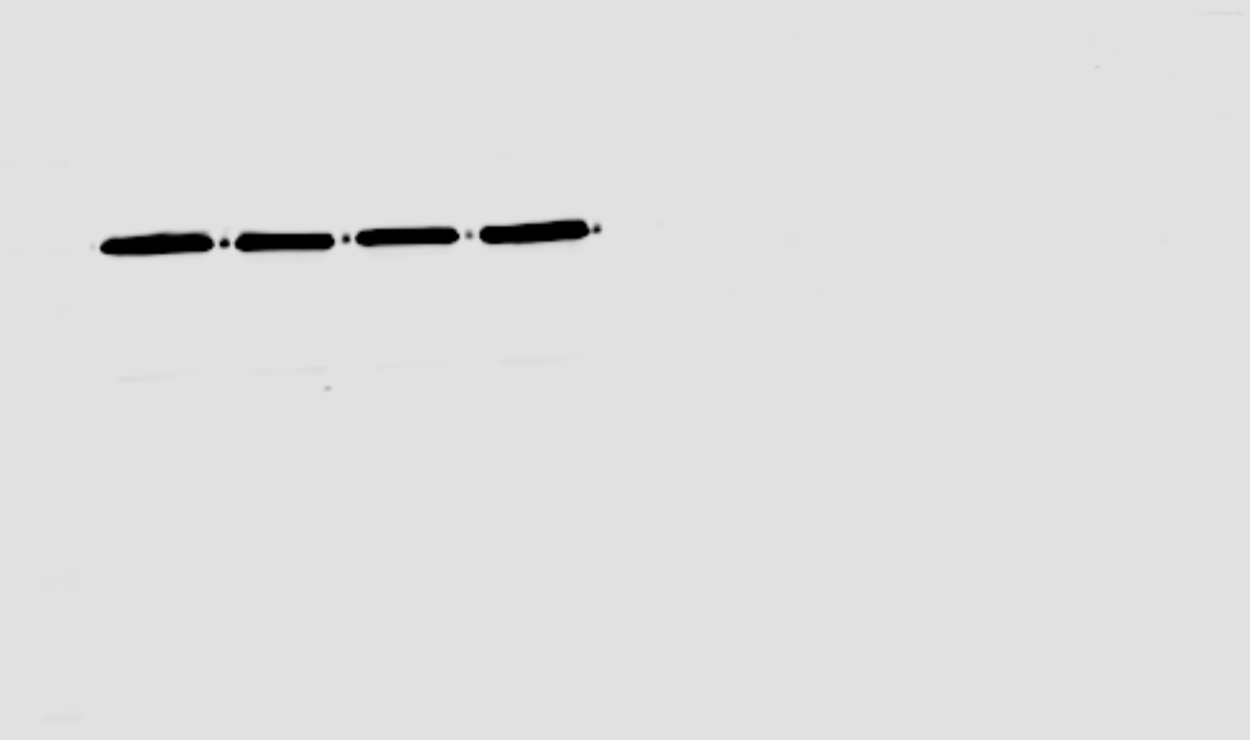

S4D_ Lane 1-3
Above ELP3_Below Tubulin

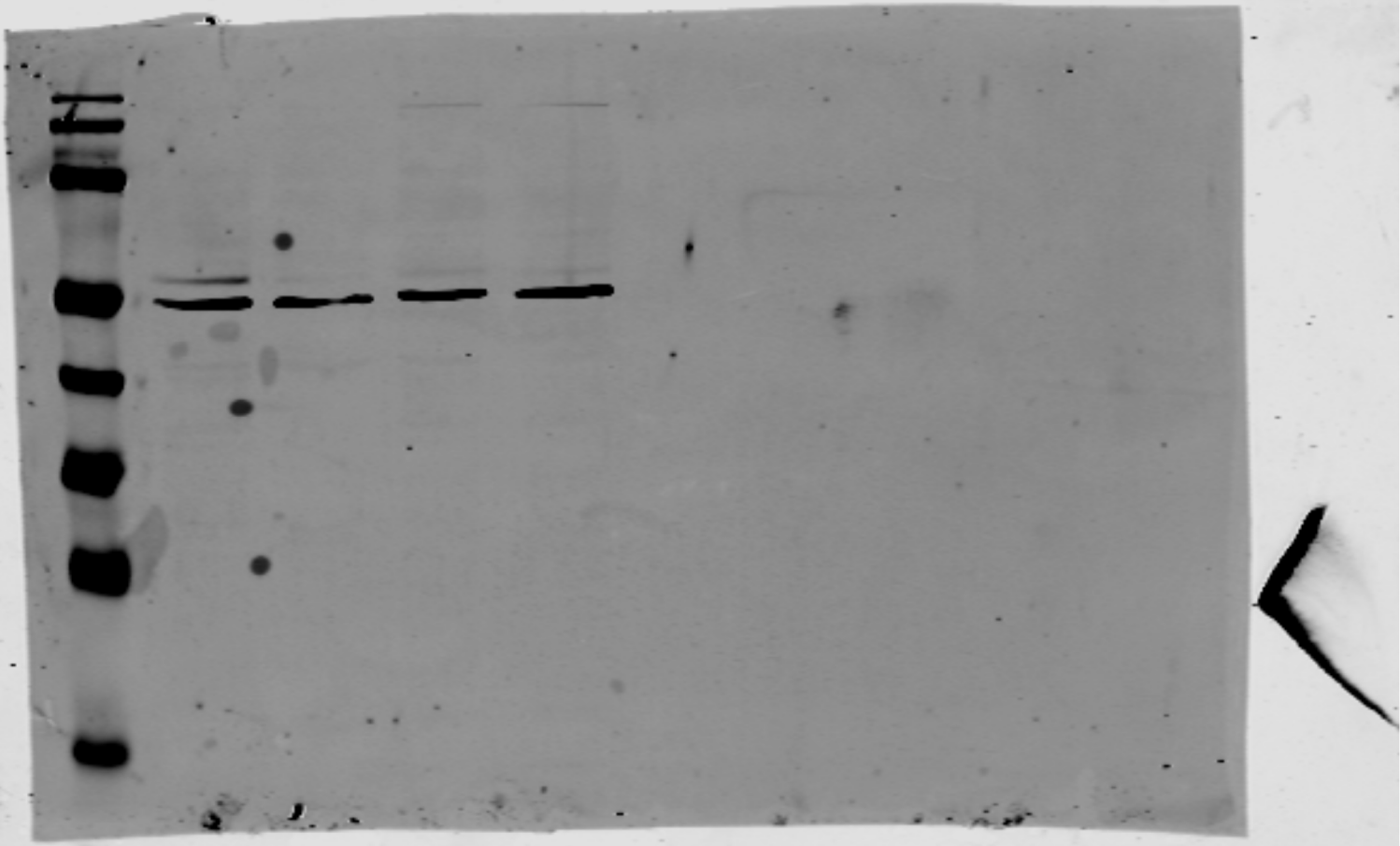

S14C- T7 HQ_IgG_HA_ All the lanes

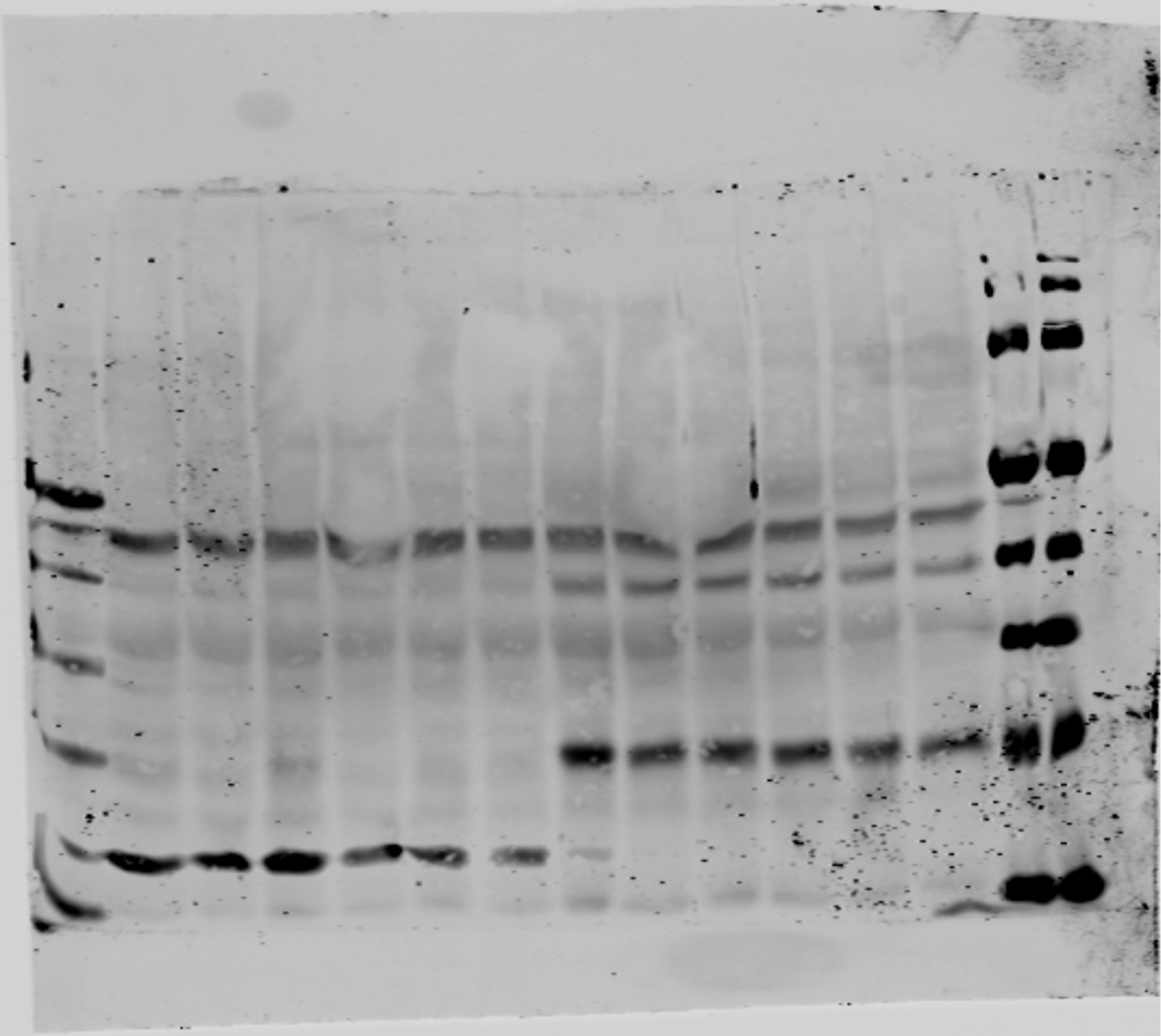

S14C- T7 HQ_Tubulin_ All the 12 lanes (MW excluded)

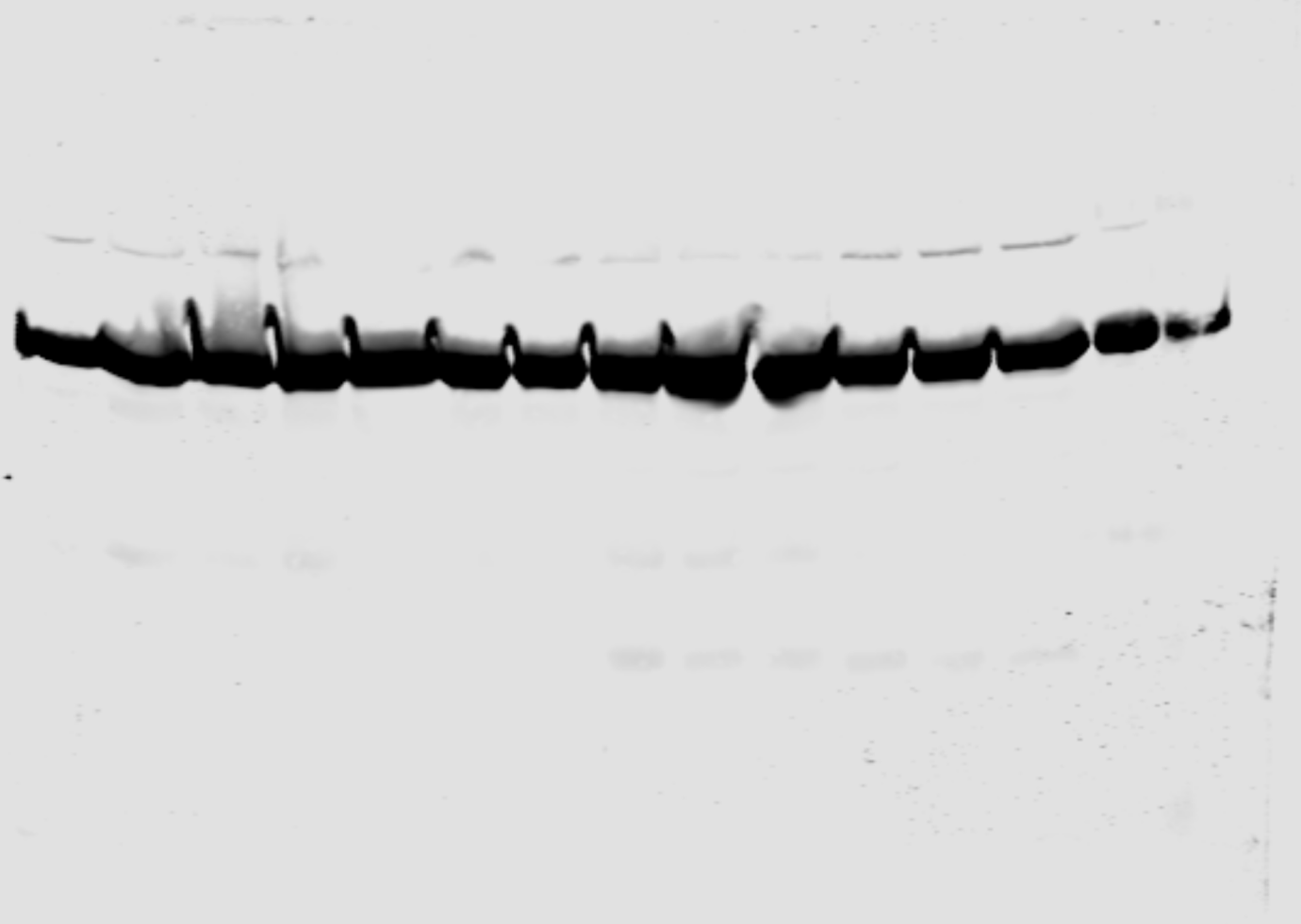

S14C- T7 QH_IgG_HA_ All the lanes

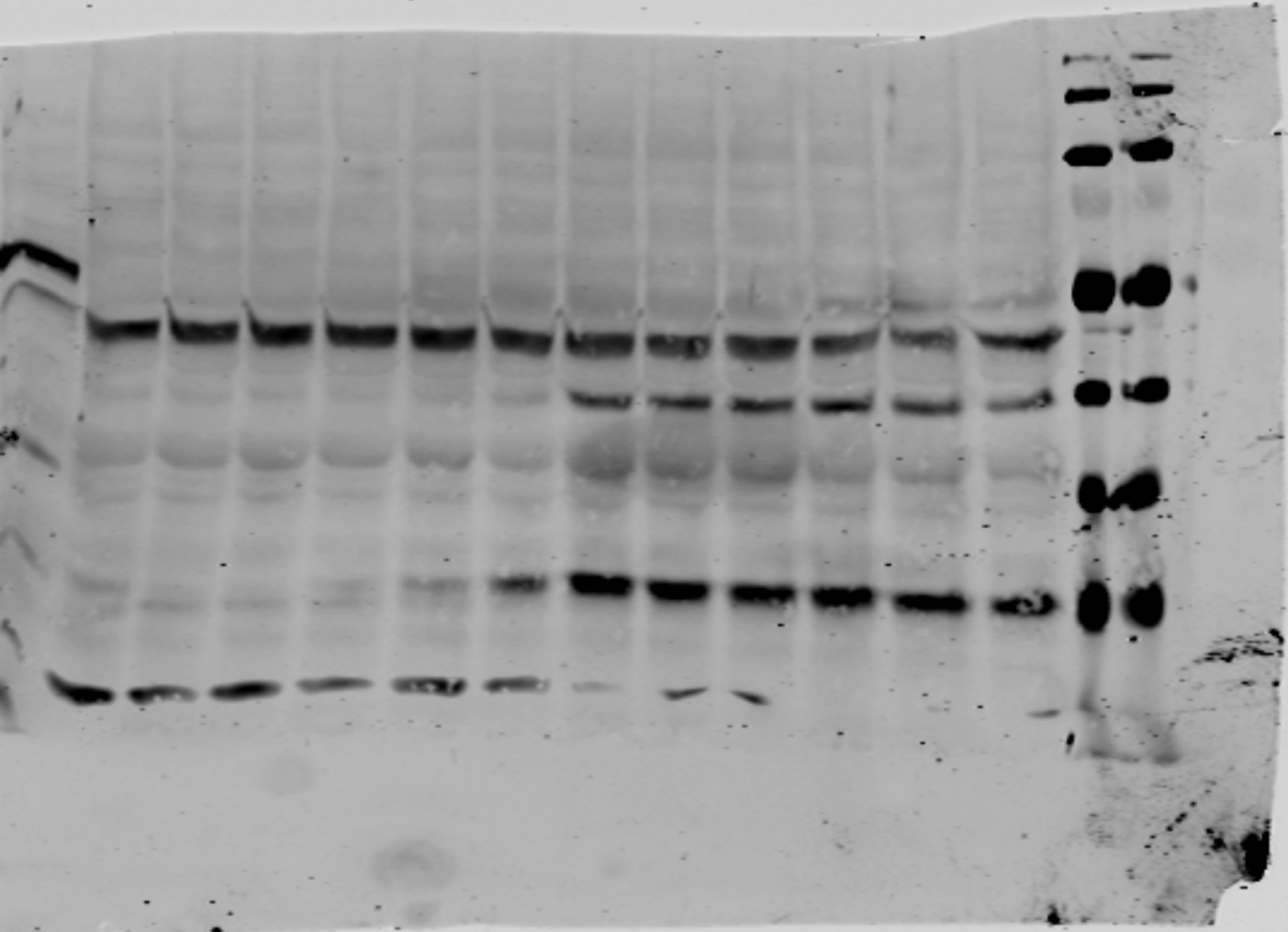

S14C- T7 QH_Tubulin_ All the lanes

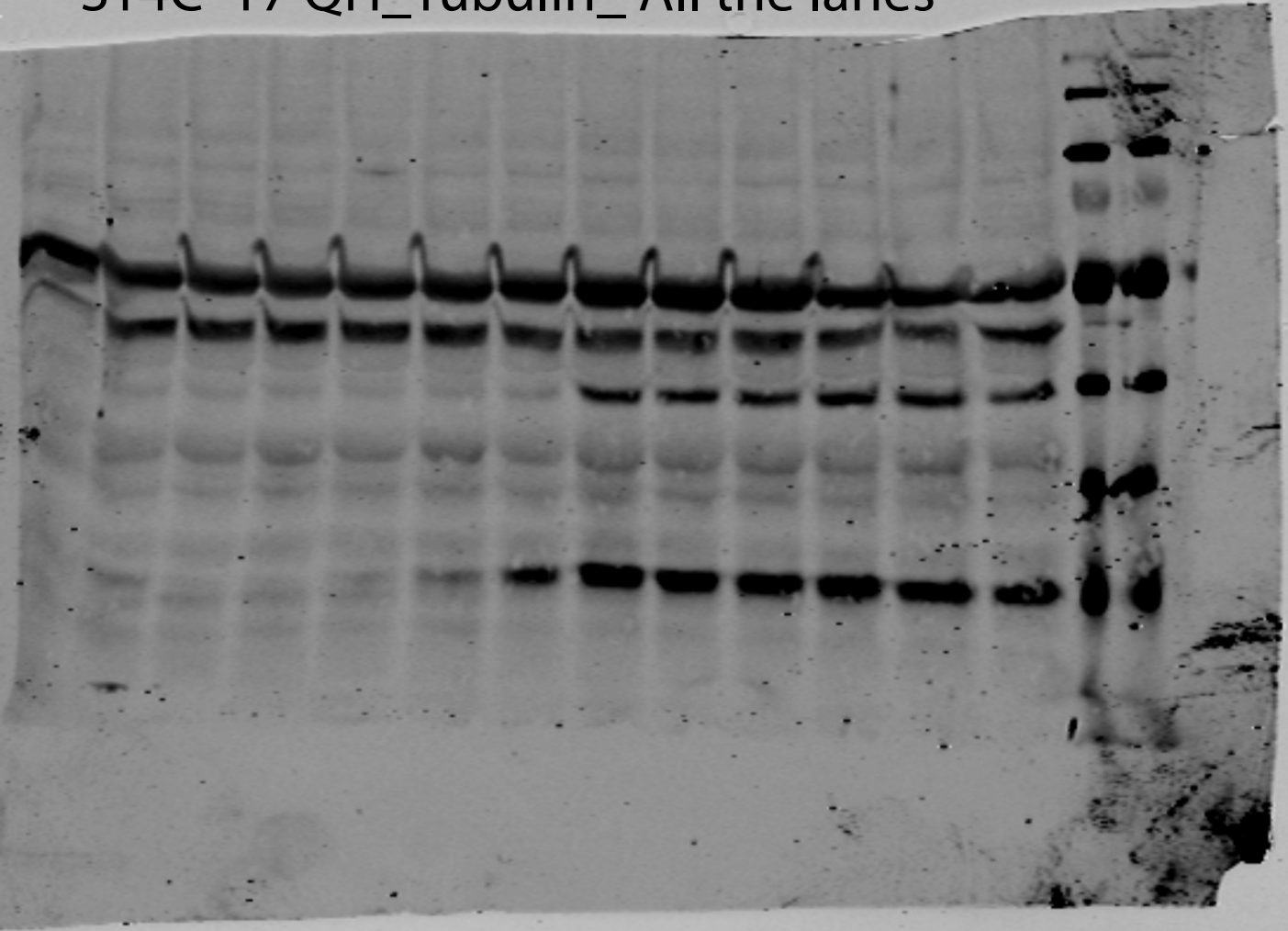

Supplement: Supplementary file 1 [file 41420_2026_3225_MOESM3_ESM.pdf]
