# Supplementary material for: Histidine to glutamine substitutants, a regulated process that impacts cell survival and suggests histidine shortage in cancer
Source: Cell Death Discov. 2026 Jun 27;12:368. doi: 10.1038/s41420-026-03225-5 (PMC13572455; doi:10.1038/s41420-026-03225-5)
Supplement: Supplementary file 2 [file 41420_2026_3225_MOESM1_ESM.docx]

# **Supplementary material**

**Histidine to glutamine substitutants, a regulated process that impacts cell survival and suggests histidine shortage in cancer**

Domenica Lovecchio ^1,7^, Pierre-René Körner ^1,7^, Lorenzo Valcanover ^1^, Onno Bleijerveld ^2^, Bart R.J. Blokhuis^3^, Abhijeet Pataskar^1^, Jasmine Montenegro Navarro ^1^, Demi Wernaart ^1^, Liesbeth Hoekman^2^, Daniil Anastasopoulos^4^, Kim Vrijland^4^, Xiaodong Feng ^1^, Julien Champagne ^1^, Karin E. de Visser ^4^, Olaf van Tellingen^5^, Frank A. Redegeld^3^, Reuven Agami ^1,6,8^

1 Division of Oncogenomics, Oncode Institute, The Netherlands Cancer Institute, Amsterdam, The Netherlands.
2 NKI Proteomics Facility, The Netherlands Cancer Institute, Amsterdam, the Netherlands.
3 Division of Pharmacology, Utrecht Institute for Pharmaceutical Sciences, Faculty of Science, Utrecht University, Utrecht, The Netherlands.
4 Division of Tumor Biology & Immunology, Oncode Institute, Netherlands Cancer Institute, Amsterdam, The Netherlands.
5 Division of Pharmacology, the Netherlands Cancer Institute, Amsterdam, the Netherlands.
6 Erasmus MC, Department of Genetics, Rotterdam University, Rotterdam, The Netherlands.
7 Contributed equally to this work.
8 Corresponding author: r.agami@nki.nl

Lead contact: Reuven Agami.

# **Supplementary figure legends**

**Supplementary Fig. S1.**

**A)** Scheme depicting filtering and validation steps for “enriched” H>Q peptides and expressed non-H>Q (WT) peptides separated by TMT and non-TMT proteomics type. Lowest part of the non-H>Q peptide shows filtering for high intensity peptides. **B-C)** Nr of enriched H>Q substitutants peptides found in TMT proteomics in three cell lines subjected to histidine shortage (HT29, -H48 h; SW480, -H 48h; PANC-1, -H 72h), with the number of the original underlying histidine codons shown in **C**. All samples were collected post 4h MG132 treatment**. D)** DIA proteomics analysis across two biological replicates per condition of PANC-1 cells: Ctrl, 50% of histidine shortage (-H 50%), 70% of histidine shortage (-H 70%), 90% of histidine shortage (-H 90%), full histidine shortage (-H), full histidine shortage (72h) followed by re-supplementation with histidine for 24 hours (-H+ H 24h), and full histidine shortage followed by re-supplementation with histidine for 48 hours (-H+ H 48h). Heatmap depicting nr of peptides expressed in both replicates. **E)** Same dataset as in (D), showing the number of peptides identified per replicate. Median values are indicated by horizontal lines. Statistical significance between groups was assessed using the unpaired two-tailed t-test (compared to Ctrl); p-values are shown as: ns non significant, *p<0.05, **p <0.002, ***p <0.001. **F**) Growth curves of PANC-1 cells subjected to varying degrees of histidine (H) shortage: Control (blue), 50% H reduction (-H 50%, black), 70% H reduction (-H 70%, green), 90% H reduction (-H 90%, orange), and complete histidine deprivation (-H, magenta). Cell confluence was monitored using the Incucyte live-cell imaging system over time (x-axis: hours), and values were normalized to the initial confluence for each condition. Data represent the mean of six biological replicates. **G)** Growth curves of PANC-1 cells under control conditions (Ctrl, blue), complete histidine deprivation (-H, magenta), or histidine deprivation followed by re-supplementation for 48 hours (-H+H 48h, black). Cell confluence was monitored over time using the Incucyte live-cell imaging system (x-axis: hours), and values were normalized to the initial confluence for each condition. Data represent the mean of six biological replicates. **H)** Boxplots depicting H>Q enrichment in Tumour and adjacent normal tissues (ANT) of CPTAC datasets [18]. The horizontal lines above the boxes indicate the p-values (Wilcoxon t-test) for the pairwise comparisons.

**Supplementary Fig. S2.**

**A)** Schematic representation of the proposed link between histidine metabolism in the tumour microenvironment and H>Q substitutants. Previously reported mechanisms are highlighted in red shading and include mast cell–derived histamine production from histidine and histamine signaling through the histamine receptor H1 (HRH1) on macrophages, associated with an immunosuppressive state and tumour progression [15]. Histidine serves as the sole precursor of histamine, linking amino acid availability to this immunoregulatory axis. Based on the findings of the present study, histidine limitation is associated with the occurrence of histidine-to-glutamine (H>Q) substituted peptides in tumours, as observed in cancer cell lines under histidine deprivation and in CPTAC patient-derived tumour datasets. **B)** Schematic depicting wild-type mast cell-proficient mice (MCPT5-Cre; Rosa26-DTA wt;het, hereafter referred to as “WT”) and mast cell-deficient mice (MCPT5-Cre;Rosa26-DTA het;het, hereafter referred to as “HET”). **C)** Histochemical staining of breast tumours derived from WT or HET mice. Sections were stained with toluidine blue and methyl green counterstain. Tumour regions are indicated by the letter “T”. **D)** Heatmaps showing log2 expression levels of histidine to glutamine (H>Q) substituted peptides detected by DIA proteomics in tumours derived from WT or HET mice. **E)** Boxplot showing the number of H>Q substituted peptides detected in WT and HET breast tumours, corresponding to the samples shown in (D). P values were calculated using the Wilcoxon test. **F)** Heatmaps showing expression levels of control proteins (e.g., GAPDH and histones), represented as log2 peptide intensities from the DIA proteomics dataset described in (D). **G)** Schematic of the HT29 cancer cell–human mast cell (HMC1.1) co-culture system. Cells were cultured at a 1:1 ratio for 4 or 7 days. **H)** Metabolomic analysis of histidine levels in fresh co-culture medium (“medium only”), medium collected after HT29–HMC1.1 co-culture, or histidine-depleted medium (“−H”). Values represent the mean ± SD of independent replicates. Non-significant values are indicated as ns; *p < 0.05, **p < 0.01, ***p < 0.001 by one-way ANOVA. **I)** Heatmap showing the number of histidine-to-glutamine (H>Q) substituted peptides and their matched wild-type peptides detected across all three replicates by DIA proteomics. Samples include HT29 cells cultured alone in co-culture medium (“HT29 only”) or HT29 cells co-cultured with HMC1.1 mast cells (“co-culture”) for 7 days. **J)** Heatmaps showing expression levels of control proteins (e.g., GAPDH and histones), represented as log2 peptide intensities from the DIA proteomics dataset described in (I).

**Supplementary Fig. S3.**

**A)** Schematic representation of tRNA glutamine (tRNA(Gln)) misalignment leading to the decoding of histidine codons. The U34 wobble position is indicated. **B)** Immunoblot showing expression levels of URM1 and Tubulin in PANC-1 cells: empty cells, cells transduced with a non-targeting sgRNA (NT), or with sgURM1 knockout constructs (URM1 KO1 and URM1 KO2). **C)** Immunoblot analysis using anti-URM1 antibody detecting URM1 and its conjugated proteins, and anti-Tubulin as a loading control, in PANC-1 cells transduced with empty vector, WT or mutant (ΔG) URM1. **D**) Number of H>Q peptides in NT, URM1 KO and URM1 ΔG in PANC-1 cells in Ctrl condition or histidine depletion (72h), followed by DIA mass spectrometry. **E)** Heatmap of DIA proteomics displayed in Figure 2B, showing now the number of the H>Q-matched WT peptides identified per condition. **F)** Heatmaps of log2 Intensity expression levels of histone proteins as log2 intensity for DIA PANC-1, as in (D-E). **G)** Boxplots of log2-transformed mean peptide intensity of H>Q peptides (red shaded) and their matched WT peptide counterparts in PANC-1 cells (-H 72h, MG-132 for 4h). The horizontal lines above the boxes indicate the p-values (Wilcoxon t-test) for the pairwise comparisons. **H)** Cell-specific quantification of protein synthesis measured by mean fluorescence intensity (MFI) of OP-Puro (Fluor 647) incorporation in PANC-1 cells under the following conditions and transduced with the specified constrcuts: no OP-Puro (NO OP-P), cycloheximide treatment (CHX), URM1 knockout (KO), non-targeting control (NT), URM1 WT, and URM1 ΔG mutant. Data represent the median of biological replicates. Statistical significance was assessed by one-way ANOVA comparing each condition to NT; ns = non significant, *p<0.05, **p <0.002, ***p <0.001.

**Supplementary Fig. S4.**

**A)** Immunoblot showing expression levels of URM1 and Tubulin in MiaPaca2 cells, transduced with a non-targeting sgRNA (NT), or with sgURM1 knockout constructs (URM1 KO1 and URM1 KO2). **B)** Heatmaps of DIA proteomics depicting the number of H>Q peptides in MiaPaca2 cells transduced with NT or URM1 KOs constructs in control or histidine depleted conditions (-H 72h, MG-132 4h). **C**) Heatmaps of proteomics depicting the number of H>Q peptides and their matched wild type (WT) counterparts in MiaPaca2 cells, same dataset as in panel (B) **D)** Immunoblot showing expression levels of ELP3 and Tubulin in PANC-1 cells: empty vector control and cells transduced with sgELP3 knockout constructs (ELP3 KO1 and ELP3 KO2). **E)** Number of H>Q peptides in NT and ELP3 KOs in PANC-1 cells in Ctrl condition or histidine depletion (72h), followed by DIA mass spectrometry. The horizontal lines above the boxes indicate the p-values (Wilcoxon t-test) for the pairwise comparisons. **F-G)** Heatmaps of expression levels of histone proteins as log2 intensity for DIA PANC-1.

**Supplementary Fig. S5.**

**A)** Venn diagram showing the overlap in unique protein names that H>Q peptides derive from, found in sgNT and URM1KO groups in the DIA PANC-1 dataset (Figure 2B; -H 72h, MG-132 4h). Green-marked peptides are lost upon URM1-KO. Blue-marked peptides are gained, grey marked peptides are shared. **B)** Barplot of Gene Ontology (GO) terms and their p-values, represented as -log10, sorted with the most significant terms at the bottom. Colors are corresponding to the group in the Venn diagram that the proteins originate from. Proteins taken from shared (grey) section of S4A Venn diagram. **C)** Barplot of Gene Ontology (GO) terms and their p-values, represented as -log10, sorted with the most significant terms at the bottom. Colors are corresponding to the group in the Venn diagram that the proteins originate from. Proteins taken from URM1KO gained (blue) section of S4A Venn diagram. **D)** Barplot of Gene Ontology (GO) terms and their p-values, represented as -log10, sorted with the most significant terms at the bottom. Colors are corresponding to the group in the Venn diagram that the proteins originate from. Proteins taken from URM1KO lost (green) section of S4A venn diagram. **E)** Venn diagram showing the overlap in unique protein names that H>Q peptides derive from, found in sgNT in histidine depletion and URM1 ΔG in the DIA PANC-1 dataset (Figure 2B; -H 72h). **F)** Barplot of Gene Ontology (GO) terms and their p-values, represented as -log10, sorted with the most significant terms at the bottom. Colors are corresponding to the group in the Venn diagram that the proteins originate from. Proteins taken from shared (grey) section of S4E Venn diagram. **G)** Barplot of Gene Ontology (GO) terms and their p-values, represented as -log10, sorted with the most significant terms at the bottom. Colors are corresponding to the group in the Venn diagram that the proteins originate from. Proteins taken from URM1 ΔG gained (dark blue) section of S4E Venn diagram. **H)** Barplot of Gene Ontology (GO) terms and their p-values, represented as -log10, sorted with the most significant terms at the bottom. Colors are corresponding to the group in the Venn diagram that the proteins originate from. Proteins taken from URM1ΔG lost (dark green) section of S4E Venn diagram.

**Supplementary Fig. S6.**

**A-B)** Boxplots presenting the log2 length of the 5' UTR, CDS, and 3' UTR for each group in PANC-1 cells, separated by line colour. Right panel of each panel is a boxplot of GC content in the CDS for each group in PANC-1 cells. **C)** Growth competition assay using PANC-1 GFP or PANC-1-mCherry cells transduced with two different URM1 knockout constructs, compared to PANC-1 cells transduced with a non-targeting (NT) control. The assay was performed under mock control conditions (Ctrl, n = 3), histidine-depleted medium (-H, n = 3), or tryptophan-depleted medium (-W, n = 6). The percentage of GFP⁺ or mCherry⁺ cells was analyzed by flow cytometry on days 4 and 7 (y axis). Data represent the median of the replicates. Statistical significance was assessed using one-way ANOVA : ns = non-significant, *p<0.05, **p < 0.002, ***p <0.001.

**Supplementary Fig. S7.**

**A-E)** Boxplots of log2-transformed mean sum intensity for the three protein groups (indicated by colour) in PANC-1, A549, HT29, and SW480 cells, subjected to DIA, DDA or TMT mass spectrometry, comparing their pairwise conditions of control and histidine depletion (48h, except for PANC-1–72h). All samples were collected post 4h MG132 treatment. The horizontal lines within the boxes represent the mean of each group. The horizontal lines above the boxes indicate the p-values (Wilcoxon t-test) for the pairwise comparisons.

**Supplementary Fig. S8.**

**A)** Barplot of top 20 Gene Ontology (GO) terms and their p-values, represented as -log10, sorted with the most significant terms at the bottom. Obtained from the top (n=100) upregulated genes after histidine depletion (-H) from the HT-29 cell line. **B)** Barplot of top 20 Gene Ontology (GO) terms and their p-values, represented as -log10, sorted with the most significant terms at the bottom. Obtained from the top (n=100) upregulated genes after tryptophan depletion (-W) from the HT-29 cell line. **C)** Barplot of top 20 Gene Ontology (GO) terms and their p-values, represented as -log10, sorted with the most significant terms at the bottom. Obtained from the bottom (n=100) downregulated genes after histidine depletion (-H) from the HT-29 cell line. **D)** Barplot of top 20 Gene Ontology (GO) terms and their p-values, represented as -log10, sorted with the most significant terms at the bottom. Obtained from the bottom (n=100) downregulated genes after W depletion (-W) from the HT-29 cell line.

**Supplementary Fig. S9.**

**A)** Barplot of top 20 Gene Ontology (GO) terms and their p-values, represented as -log10, sorted with the most significant terms at the bottom. Obtained from the top (n=100) upregulated proteins after H depletions from the HT29 cell line. **B)** Barplot of top 20 Gene Ontology (GO) terms and their p-values, represented as -log10, sorted with the most significant terms at the bottom. Obtained from the bottom (n=100) downregulated proteins after H depletion (-H) from the HT29 cell line. **C)** Barplot of top 20 Gene Ontology (GO) terms and their p-values, represented as -log10, sorted with the most significant terms at the bottom. Obtained from the top (n=100) upregulated proteins after H depletion (-H) from the PANC-1 cell line. **D)** Barplot of top 20 Gene Ontology (GO) terms and their p-values, represented as -log10, sorted with the most significant terms at the bottom. Obtained from the bottom (n=100) downregulated proteins after H depletion (-H) from the PANC-1 cell line.

**Supplementary Fig. S10.**

**A**) Barplot of top 20 Gene Ontology (GO) terms and their p-values, represented as -log10, sorted with the most significant terms at the bottom. Obtained from the (n=414) H>Q proteins from the HT-29 cell line. **B)** Barplot of top 20 Gene Ontology (GO) terms and their p-values, represented as -log10, sorted with the most significant terms at the bottom. Obtained from the (n= 415) non-H>Q top proteins from the HT-29 cell line. **C)** Barplot of top 20 Gene Ontology (GO) terms and their p-values, represented as -log10, sorted with the most significant terms at the bottom. Obtained from the (n=116) H>Q proteins from the PANC-1 cell line. **D)** Barplot of top 20 Gene Ontology (GO) terms and their p-values, represented as -log10, sorted with the most significant terms at the bottom. Obtained from the (n=117) non-H>Q top proteins from the PANC-1 cell line.

**Supplementary Fig. S11.**

**A-E)** Boxplots presenting the log2 length of the 5' UTR, CDS, and 3' UTR for each group (depleted,depleted top and H>Q) in the datasets of Supplementary Fig. S7, separated by line colour. Right panel of each panel is a boxplot of GC content in the CDS for each group.

**Supplementary Fig. S12.**

**A)** Heatmap of expression levels of histone proteins as log2 intensity for PANC-1 TMT dataset. X-axis represents the condition, non-treated (Ctrl), histidine depletion medium (-H) for 72 hours and MG132 for 4 hours. **B)** Heatmap of expression levels of histone proteins as log2 intensity for PANC-1- and MiaPaca2 DIA dataset. X-axis represents the condition, non-treated (Ctrl), histidine depletion medium (-H) for 48 hours and MG-132 for 4 hours. **C)** Heatmap of expression levels of histone proteins as log2 intensity for A549 DIA dataset. X-axis represents the condition, non-treated (Ctrl), histidine depletion medium (-H) for 48 hours and MG132 for 4 hours, and tryptophan depletion medium (-W).

**Supplementary Fig. S13.**

**A)** Heatmap of expression levels of histone proteins as log2 intensity for HT29 DDA dataset. X-axis represents the condition, non-treated (Ctrl), histidine depletion medium (-H) for 48 hours and MG132 for 4 hours. **B)** Heatmap of expression levels of histone proteins as TMT-ratio for HT29 TMT dataset. X-axis represents the condition, non-treated (Ctrl), histidine depletion medium (-H) for 48 hours and MG-132 for 4 hours. **C)** Heatmap of expression levels of histone proteins as TMT-ratio for SW480 TMT dataset. X-axis represents the condition, non treated (Ctrl), histidine depletion medium (-H) for 48 hours and MG-132 for 4 hours.

**Supplementary Fig. S14.**

**A)** Heatmap of differences in nucleotide frequency in a window of three nucleotides before or after tryptophan codon W in the A549 DIA dataset. **B**) Heatmap of differences in amino acid frequency in a window of ten amino acid before or after histidine codons in the A549 DIA dataset. **C**) Schematic of the reporter construct (T7) above the western blots. Western blots anti-HA depicting HA-IP efficiency with 20µL of the total sample before immunoprecipitation and then of the supernatant depleted of HA after the incubation with anti-HA beads. **D**) Comparison of mRNA expression levels of the T7 QQ, T7 HQ, and T7 QH reporters in MD55 cells under untreated (+H) or full histidine depletion (-H) conditions. Two sets of primers used for detection are indicated. Technical replicates are shown in matching colors, while biological replicates are represented by two different colors (black and turquoise). Statistical significance was determined by ordinary one-way ANOVA; ns = non-significant; *p<0.05, **p <0.002, ***p <0.001. **E)** Arginine IP reporter schematic. Heatmaps showing the log2 Intensity of IP reporter peptides expression in HT29 cells subjected to histidine shortage for 48 h and 4h of M-132 treatment, measured by mass spectrometry. On x-axis displayed are the arginine codons that followed histidine in the reporter as well as the conditions of untreated (+H) or full histidine depletion (-H) conditions.

**Supplementary Fig. S15.**

**A)** Heatmap displaying the fraction (0 to 1 on the y=axis) of histidine codon (CAC, CAT) distribution per transcript in the reference proteome on the left two lanes (x-axis) and the histidine codon with their following nucleotide (+4) in the further lanes (x-axis). **B)** Boxplots displaying the fraction (0 to 1 on the y=axis) of nucleotides (+4, nucleotide indicated by color) following histidine codons (CAC or CAT on x-axis). P values are calculated by pairwise Wilcoxon-test (*** starts indicating highly significant < 0.05). **C)** Heatmaps of differences in nucleotide frequency in a window of three nucleotides before or after histidine codons in the A549 DIA dataset. Displayed are the heatmaps of all H>Q peptides, the top WT peptides (non-H>Q), and histidine codons CAC and CAT shown separately. The number of histidine codons the analysis is based on is indicated above each heatmap (n=). **D)** Heatmaps of differences in nucleotide frequency in a window of three nucleotides before or after histidine codons in the HT29 DDA dataset. Displayed are the heatmaps of all H>Q peptides, the top WT peptides (non-H>Q), and histidine codons CAC and CAT shown separately. The number of histidine codons the analysis is based on is indicated above each heatmap (n=).

**Supplementary Fig. S16.**

**A)** Heatmaps of differences in nucleotide frequency in a window of three nucleotides before or after histidine codons in the HT29 TMT dataset. Displayed are the heatmaps of all H>Q peptides, the top WT peptides (non-H>Q), and histidine codons CAC and CAT shown separately. The number of histidine codons the analysis is based on is indicated above each heatmap (n=). **B)** Heatmaps of differences in nucleotide frequency in a window of three nucleotides before or after histidine codons in the AsPc-1 TMT dataset. Displayed are the heatmaps of all H>Q peptides, the top WT peptides (non-H>Q), and histidine codons CAC and CAT shown separately.. The number of histidine codons the analysis is based on is indicated above each heatmap (n=). **C)** Heatmaps of differences in nucleotide frequency in a window of three nucleotides before or after histidine codons in the PANC-1 DIA dataset. Displayed are the heatmaps of all H>Q peptides, the top WT peptides (non-H>Q) , and histidine codons CAC and CAT shown separately. The number of histidine codons the analysis is based on is indicated above each heatmap (n=). **D)** Heatmaps of differences in nucleotide frequency in a window of three nucleotides before or after histidine codons in the PANC-1 TMT dataset. Displayed are the heatmaps of all H>Q peptides, the top WT peptides (non-H>Q), and histidine codons CAC and CAT shown separately. The number of histidine codons the analysis is based on is indicated above each heatmap (n=). **E)** Heatmaps of differences in nucleotide frequency in a window of three nucleotides before or after histidine codons in the SW480 TMT dataset. Displayed are the heatmaps of all H>Q peptides, the top WT peptides (non-H>Q), and histidine codons CAC and CAT shown separately. The number of histidine codons the analysis is based on is indicated above each heatmap (n=).

**Supplementary Fig. S17.**

**A-E)** Violin plot of the CACA/CACC ratio for the two protein groups (color-coded) in the the A549 DIA dataset (**A**), HT29 DDA dataset (**B**), HT29 TMT dataset (**C**), in the PANC-1 TMT dataset (**D**) and in the SW480 TMT dataset (**E**). Horizontal lines show group means. P-values by Wilcoxon t-test for the pairwise comparisons. **F)** Bar plot of Gene Ontology (GO) terms with −log₁₀ p-values, sorted with the most significant terms at the bottom. Derived from the highest decile of H>Q CACA/CACC proteins (h). **G**) Heatmaps of differences in nucleotide frequency on position +4 after Histidine (H= 0, +3) showing the groups NT, URM1 KO, and URM1 ΔG, compared against expressed wild type, dataset as in Figure 2B. Shown are CAC+CAT, and the two codons split.
